# Therapeutic targeting of STING-IL6/STAT3 axis to inhibit osteoclastic niche formation and breast cancer bone metastasis

**DOI:** 10.1038/s41420-025-02776-3

**Published:** 2025-10-24

**Authors:** Chen Zhao, Pengcheng Liu, Keyu Kong, Xuzhuo Chen, Junxiang Wu, Wen Wu, Xiaoqing Wang, Lei Wang

**Affiliations:** 1 Department of Orthopedics, Shanghai Key Laboratory of Orthopedics Implant, The Ninth People’s Hospital, Shanghai Jiao Tong University School of Medicine, Shanghai, China; 2 Department of Orthopedics, Shanghai General Hospital, Shanghai Jiao Tong University School of Medicine, Shanghai, China; 3 Department of Oral Surgery, Shanghai Key Laboratory of Stomatology & Shanghai Research Institute of Stomatology, National Clinical Research Center for Oral Diseases, Shanghai Ninth People’s Hospital, College of Stomatology, Shanghai Jiao Tong University School of Medicine, Shanghai, China

**Keywords:** Bone cancer, Cancer microenvironment

## Abstract

Despite numerous studies on tumor bone metastasis, little emphasis has been placed on the role of the formation of the osteoclastic premetastatic niche (OPN), and related genetic intervention targets are relatively scarce. Our data confirm the promoting effect of OPN formation on bone metastasis and demonstrate the existence of a vicious cycle between osteoclast differentiation and breast tumor cell proliferation through in vitro cell experiments. Moreover, we show that regulating Stimulator of Interferon Genes (STING) can break this cycle. However, both in vivo and in vitro experiments further indicate that STING intervention in OPN formation and breast tumor bone metastasis depends on IL6/STAT3, with a significant discount in the presence of IL-6 activation. In summary, these data not only highlight the important role of OPN but also emphasize STING-IL6/STAT3 as a crucial intervention target.

## Highlights

- OPN formation drives breast cancer bone metastasis by linking osteoclast activity and tumor proliferation.
- STING modulation disrupts this process but is dependent on IL-6/STAT3 signaling.
- IL-6 activation significantly diminishes STING’s therapeutic potential.
- The STING-IL6/STAT3 axis emerges as a critical target for breast cancer bone metastasis interventions.

## Introduction

The bone is the third most common site for metastasis across a broad spectrum of solid tumors [1–4]. The diagnosis of bone metastasis often relies on the emergence of severe symptoms, including pain and pathological fractures. These symptoms arise due to a vicious cycle involving metastatic cells, bone-resorbing cells, and osteoclasts [5, 6]. However, once tumor cells metastasize to the bone, both treatment and prognosis become significantly challenging. Therefore, identifying the factors that predispose patients to bone metastasis at an early stage, potentially before tumor dissemination, could hold substantial clinical utility for prevention strategies.

Bone metastasis is a common and devastating complication of various solid tumors, including breast [7–9], prostate [10], lung [11], and multiple myeloma [12], often leading to severe skeletal-related events and poor prognosis. Although these tumors share a predilection for the bone microenvironment, their metastatic processes display both overlapping and distinct biological features. For instance, breast cancer and lung cancer cells preferentially colonize bone by engaging specific signaling pathways such as CXCL5/CXCR2, JAK1/STAT3, and EZH2-integrin β1-FAK axes, which promote tumor proliferation and osteolytic or osteoclastic lesions, respectively [8, 11, 13]. Despite this heterogeneity, osteoclasts and their precursors emerge as pivotal components in establishing a conducive premetastatic niche across different tumor types, underscoring the importance of osteoclast-driven bone remodeling and immune modulation in metastasis progression [14, 15].

The formation of a premetastatic niche for osteoclastic bone metastasis serves as an early warning sign, suggesting that the process of tumor bone metastasis begins prior to the secretion of osteoclast-stimulating factors by pre-osteoclasts following tumor cell colonization within the bones [16]. Therefore, targeting osteoclasts for diagnosis and treatment may need to occur at the early stages of tumor detection. While key cells of the premetastatic niche for bone metastasis have been identified [14], further research is required to elucidate the crucial systemic gene regulatory mechanisms and their upstream and downstream regulatory relationships.

STING, or Stimulator of Interferon Genes, is a pivotal signaling molecule in the immune system, sensing DNA damage and viral infections within cells to initiate immune responses to counteract these threats [17, 18]. STING is primarily localized in the endoplasmic reticulum within cells and becomes activated upon cellular exposure to DNA viruses or DNA damage. Upon activation, STING promotes the production of interferons and other inflammatory factors, thereby triggering the activation of immune cells and inflammatory responses [19]. Once activated by its cyclic dinucleotide ligand or gain-of-function mutations resulting in constitutive ER exit, STING translocates from the endoplasmic reticulum to the Golgi apparatus, where it undergoes phosphorylation by TBK1 [18, 20]. Previous studies have also demonstrated a significant regulatory role of STING in skeletal immune diseases [21–23]. In cancer research, STING agonizts have shown potential in inhibiting cancer-related bone pain [24].

Despite increasing recognition of the importance of the premetastatic niche in facilitating bone metastasis, the concept of an osteoclastic premetastatic niche (OPN) remains underexplored. Moreover, while STING is well known for its role in initiating innate immune responses and modulating skeletal inflammation, its potential function in regulating OPN formation remains unclear. The extent to which STING-driven immune modulation influences osteoclast differentiation, niche priming, and downstream metastatic behavior has not been elucidated. Addressing this knowledge gap could provide new insights into early intervention strategies for bone metastasis, targeting both immune and skeletal components before irreversible metastatic colonization occurs.

Based on these findings, we speculate that the systemic regulatory effects of STING on the immune system and its stabilizing effects on bone metabolism balance could be applicable to regulating premetastatic niche formation involving osteoclasts. A series of explorations and studies have been conducted around breast cancer and breast cancer bone metastasis models. Breast cancer serves as an ideal model to investigate bone metastasis due to its high incidence of skeletal dissemination and well-established cell line models that recapitulate many aspects of human disease. The availability of advanced imaging, genetic manipulation tools, and clinically relevant cell lines makes breast cancer particularly suitable for dissecting the molecular and cellular mechanisms governing premetastatic niche formation, osteoclast-tumor interactions, and metastatic colonization. Therefore, focusing on breast cancer allows for a comprehensive exploration of the bone metastatic process, with findings potentially translatable to other malignancies with bone involvement.

## Results

### Formation of OPN facilitates bone metastasis in breast cancer

In the initial part of the study, we aimed to determine if tumors could create a pre-osteoclast metastasis microenvironment conducive to tumor cell dissemination into the bone, known as an OPN, before distant bone metastasis occurs. To achieve this goal, we collected conditioned media (CM) from MDA-MB-231 and 4T1 cells, two cell lines that are highly metastatic and invasive and are often used to study distant metastasis of breast cancer. Based on previous research, we intraperitoneally injected this CM collected from cultured cells into mice to simulate the influence of tumor cells on the bone microenvironment prior to metastases [14].

**Fig. 1:**
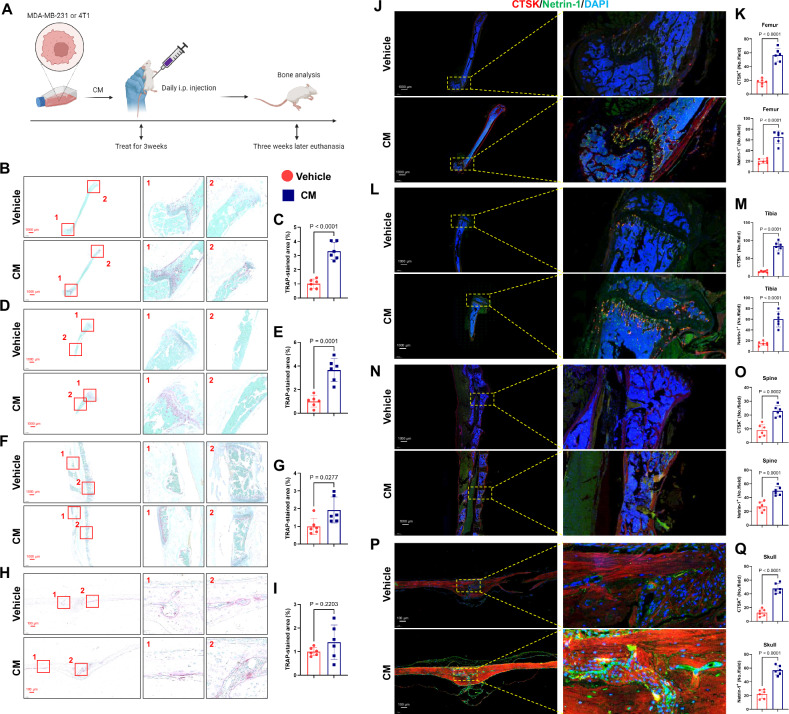
Conditioned medium (CM) of cancer cells promoted osteoclastic pre-metastatic niche formation. **A** Experimental design for the collection and injection of CM. **B**–**I** TRAP staining (from top to bottom: femur, tibia, spine, and skull). Scale bars, 1000 µm. On the right is a quantitative analysis of the number of TRAP^+^ cells per bone marrow area. *n* = 6 per group. **J**–**Q** Representative images of immunofluorescence staining of CTSK (red) and Netrin-1 (green) in the femur, tibia, spine, and skull (left, from top to bottom) and quantitative analysis (right). Scale bars, 1000 µm and 100 µm. *n* = 6 per group. Data are presented as mean ± SD. Significance levels are indicated on top of each comparison. Comparisons between the two groups were performed using an unpaired two-sided Student’s *t*-test.

Furthermore, we administered these injections continuously for three weeks to ensure the maximal impact of premetastatic tumor cells on the bone microenvironment (Fig. 1A). Our results showed that compared with the vehicle group, after three consecutive weeks of CM injection, common tumor bone metastasis sites, such as the distal femur, proximal tibia, and spine, exhibited a significant enhancement in osteoclast differentiation, as indicated by an increase in the range and degree of tartrate-resistant acid phosphatase (TRAP) staining positivity (Fig. 1B–G). However, no significant differences were found in pro-osteoclast differentiation in the skull, consistent with the predilection sites for breast cancer metastasis (Fig. 1H, I). This finding indicates that tumors exert a positive influence on the formation of the OPN in situ before metastasis.

Netrin-1, upregulated in cancers as a pro-tumoral mechanism, is re-expressed in both cancer cells and the tumor microenvironment in many human neoplasms [25–27]. Targeting Netrin-1 can effectively curb the epithelial-to-mesenchymal transition of tumors and improve their response to chemotherapy [27]. Moreover, osteoclast-derived Netrin-1 also plays an important role in the induction of nerve growth and mediating pain [28]. Therefore, based on the above studies, we aimed to clarify whether Netrin-1 derived from osteoclasts attracts primary tumors to bone metastasis during the formation of an OPN. Cathepsin K (CTSK) is a protease mainly found in the skeletal system. It is expressed in mature bone cells, such as osteoclasts, facilitating bone remodeling and metabolism. CTSK degrades bone matrix proteins, such as collagen and osteotin, thereby promoting bone resorption. Therefore, it is often used to determine osteoclast expression levels. We observed that high CTSK expression corresponded to enhanced Netrin-1 expression in osteoclasts after the OPN formation (Fig. 1J–Q).

**Fig. 2:**
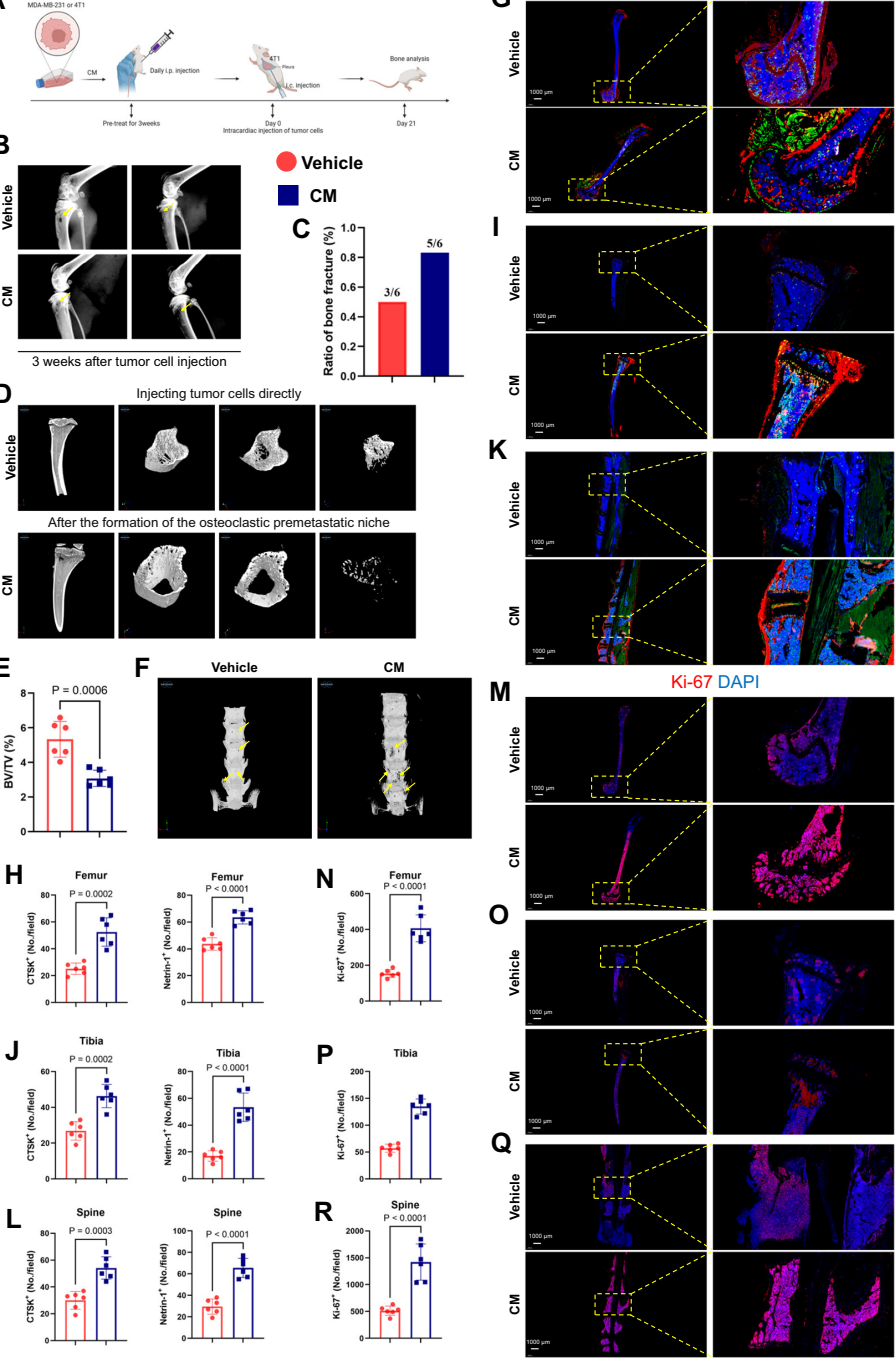
Formation of osteoclastic pre-metastatic niche promotes bone metastasis. **A** Experimental design for the injection of tumor cells after the formation of a pre-metastatic microenvironment. **B** Representative radiographs of long bones bearing tumors from mice treated with vehicle or CM. The yellow arrows indicate sites of bone destruction. **C** Ratio of bone fractures on day 21 after injection of cancer cells between the vehicle and CM treatment groups. *n* = 6 per group. **D** Micro-CT images showing trabecular and cortical bone destruction in the proximal tibia. **E** Morphometric quantification of cortical bone micro-CT images with analysis of bone volume fraction (BV/TV) from mice treated with vehicle or CM. *n* = 6 per group. **F** Micro-CT images showing bone destruction on the surface of the vertebral bodies. The yellow arrows indicate sites of bone destruction. **G**–**L** Representative images of immunofluorescence staining of CTSK (red) and Netrin-1 (green) in the femur, tibia, and spine (right, from top to bottom) and quantitative analysis (left). Scale bars, 1000 µm. *n* = 6 per group. **M**–**R** Representative immunofluorescent images (**M**, **O**, **Q**) and quantification (**N**, **P**, **R**) of proliferative tumor cells in the femur, tibia, and spine (*n* = 6 mice), as determined by the presence of Ki-67^+^. Scale bars, 1000 µm. Data are presented as mean ± SD. Significance levels are indicated on top of each comparison. Comparisons between the two groups were performed using an unpaired two-sided Student’s *t*-test.

Next, we confirmed the substantial impact of this osteoclast premetastatic microenvironment on bone metastasis. Consistent with the above, we first formed an OPN through intraperitoneal injection of conditioned medium (CM) and then achieved blood-source seeding of tumors through intracardiac injection or tail vein injection of 4T1 cells (Fig. 2A). We assessed tumor-induced osteolytic bone destruction using radiography (Fig. 2B). Compared with the control group, in which tumor cells were injected before premetastatic niche formation, the established OPN significantly enhanced tumor metastasis to the bone. This was manifested radiographically as more pronounced bone destruction and a higher incidence of bone erosion at a consistent euthanasia time point (Fig. 2B, C).

For a more detailed analysis of the significant impact of bone metastasis on the bone microstructure, we conducted micro-CT analysis and three-dimensional reconstruction of the proximal tibia, the primary site of metastatic bone tumor burden (Fig. 2D). We extensively explored cortical and trabecular aspects from multiple perspectives to gain insights into their effects on bone architecture. The results indicated that compared to the control group, the formation of the premetastatic niche prior to metastasis enhanced cortical destruction, medullary loss, and alterations in the ratio of trabecular bone volume to total volume (BV/TV) and connectivity density (Fig. 2D, E). Additionally, the spine, another common site of bone metastasis, showed increased burden due to the premetastatic microenvironment (Fig. 2F). Subsequently, immunofluorescence (IF) experiments revealed that, along with significant bone destruction, the sites of bone metastasis exhibited a more pronounced release of Netrin-1 originating from osteoclasts (Fig. 2G–L). Lastly, the presence of Ki-67-positive cells also suggested a robust promotive effect of the OPN on enhanced cellular proliferation and vitality (Fig. 2M–R). Therefore, before a tumor has substantial metastasis, an environment that attracts tumor metastasis is formed in the bone, such as a lighthouse lighting up a cruise ship on the sea. In summary, these findings demonstrate that before tumor cell metastasis, the formation of an OPN provides potent chemotactic ability for tumor migration to the bone and subsequently enhances the osteolytic capacity of metastasized tumors.

### STING activation suppresses the formation of OPNs

**Fig. 3:**
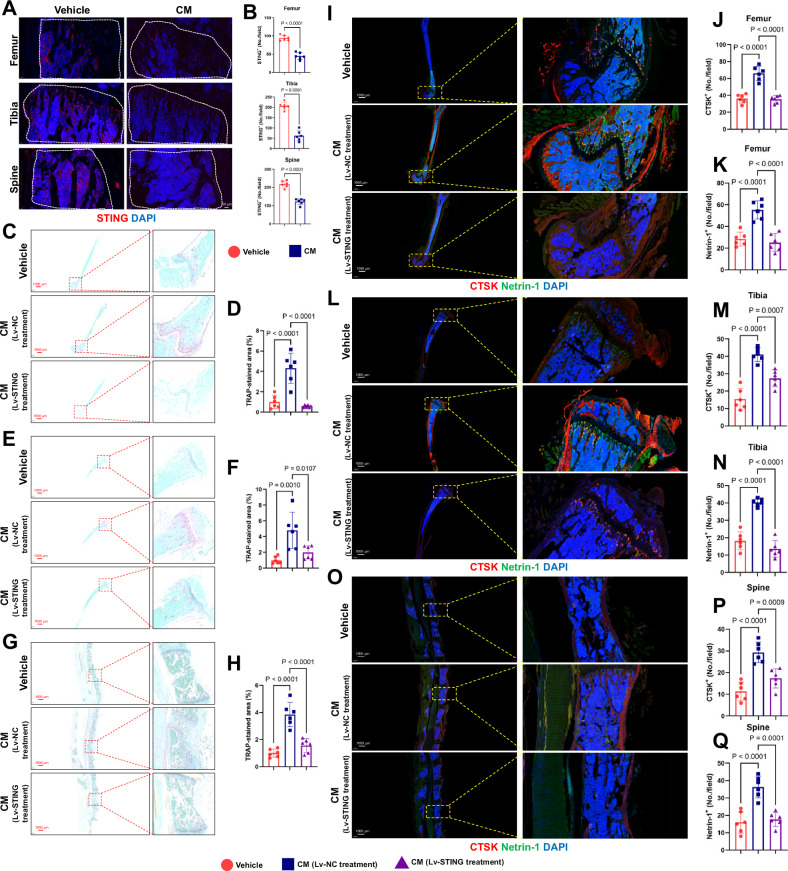
STING activation suppresses the formation of OPNs. **A**, **B** Representative images of IF staining of STING (red) in the femur, tibia, and spine (left, from top to bottom) and quantitative analysis (right). The statistical area is within the white line. Scale bars, 50 µm. *n* = 6 per group. **C**–**H** TRAP staining (from top to bottom: femur, tibia, and spine) from the Vehicle group, CM group derived from infection with empty vector virus, and CM group derived from infection with STING-expressing virus. Scale bars, 1000 µm. On the right is a quantitative analysis of the number of TRAP^+^ cells per bone marrow area. *n* = 6 per group. **I**–**Q** Representative images of IF staining of CTSK (red) and Netrin-1 (green) in the femur, tibia, and spine (left, from top to bottom) and quantitative analysis (right). Scale bars, 1000 µm. *n* = 6 per group. Data are presented as mean ± SD. Significance levels are indicated on top of each comparison. *P* values were calculated using a one-way ANOVA with a multiple comparisons test.

The STING pathway triggers innate immune responses to cytosolic dsDNA in cancer cells [29]. Previous studies have suggested its role in tumor bone destruction and the improvement of cancer pain [24]. However, its potential impact on the early stages of tumor bone metastasis through its suppressive effect on the formation of a premetastatic niche remains unexplored. Given the significant potential of STING in both tumor and bone metastasis, we aimed to address this gap. By intraperitoneally injecting CM for three weeks, we found that the expression levels of STING were significantly downregulated in the intraosseous microenvironment of the femur, tibia, and spine compared to the control group (Fig. 3A, B). This observation suggests the potential of STING to regulate OPN formation. To further investigate the role of STING in OPN formation induced by tumor cells, we overactivated STING in MDA-MB-231 and 4T1 cells via lentiviral infection. Subsequently, we collected CM from cells overexpressing STING and cells infected with an empty vector lentivirus. The results of TRAP staining indicated that CM derived from cells overexpressing STING was less effective in promoting the formation of an OPN than CM from cells infected with the control vector (Fig. 3C–H). It is noteworthy that while CM derived from STING-expressing cells in the skull could rescue the enhancement of TRAP staining, CM derived from cells transfected with empty vector viruses was also unable to promote osteoclast differentiation effectively compared to the vehicle (Supplementary Fig. 1A). Fluorescent double staining results also suggested that overexpression of STING within tumor cells effectively rescued the formation of the premetastatic microenvironment and the high expression of metastatic factors in tumor cell-derived CM (Fig. 3I–Q). Similarly, this protective effect, evident in the femur, tibia, and spine, was not observed in the skull (Supplementary Fig. 1B). Based on these results, we speculate that the effective activation and overexpression of STING in tumor cells can inhibit the formation of a pre-metastatic microenvironment in distant bones before metastasis.

### The vicious cycle: STING regulation of breast cancer bone metastasis

**Fig. 4:**
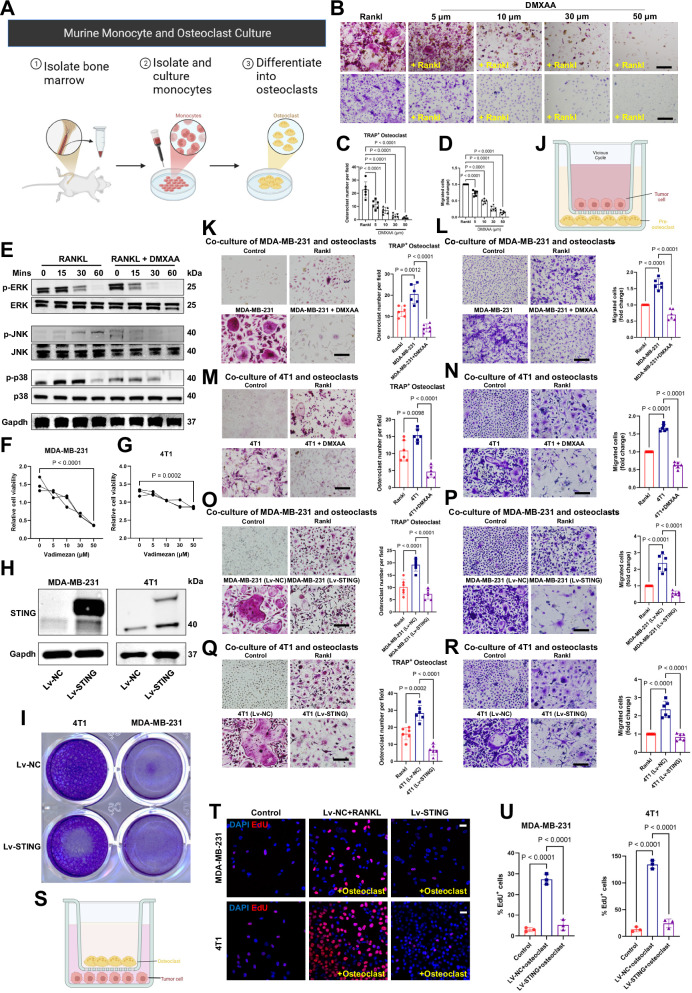
The vicious cycle: STING regulation of breast cancer bone metastasis. **A** Experimental design for isolation of murine monocytes and culture of osteoclasts. **B**–**D** Treatment with DMXAA inhibits RANKL-induced migration and osteoclast differentiation. Bone marrow-derived macrophages (BMDMs) from mice were used to generate osteoclasts by stimulation with 30 ng·mL^−1^ M-CSF and 100 ng·mL^−1^ RANKL. Representative images of cells from the migration assay (crystal violet staining) (bottom) and osteoclast differentiation assay (TRAP staining) (top), and the corresponding quantitative analysis (**C**, **D**). Scale bars, 0.1 cm. *n* = 6 experiments. **E** Western blot showing p-ERK, p-JNK, and p-p38 in BMDM treated with RANKL (100 ng/mL) or RANKL (100 ng/ mL) together with DMXAA at different time points. **F**, **G** Cell viability of MDA-MB-231 and 4T1 after treatment with different concentrations of DMXAA. *n* = 3 experiments. **H** Western blot showing STING expression in MDA-MB-231 and 4T1 treated with lentivirus expressing STING. **I** Representative images of cells post-infection with STING-expressing virus from the migration assay (stained with crystal violet). **J** Model of co-culture of breast cancer cells with preosteoclasts. Murine BMDM preosteoclasts were seeded into the lower chamber plates. Breast cancer cells were seeded into the cell culture inserts in the upper chamber plates and treated with DMXAA or lentivirus expressing STING. **K**–**R** Representative images of cells from the migration assay (crystal violet staining) (right) and osteoclast differentiation assay (TRAP staining) (left), along with corresponding quantitative analysis. The upper layer of tumor cells was either activated for STING via DMXAA treatment or pre-infected with lentivirus expressing STING. Scale bars, 0.1 cm. *n* = 6 experiments. **S** Model of co-culture of breast cancer cells with preosteoclasts, differing from (**J**) by exchanging the positions of the upper and lower cell layers. **T** EdU labeling analysis of the influence of osteoclast differentiation on the proliferation of lower-layer tumor cells, accompanied by corresponding quantitative analysis (**U**). Scale bars, 25 µm. *n* = 3 per group. Data are presented as mean ± SD. Significance levels are indicated on top of each comparison. *P* values were calculated using a one-way or two-way ANOVA with a multiple comparisons test.

To further understand the regulatory role of STING in tumor bone metastasis and destruction, we isolated and cultured primary mouse osteoclasts and conducted a series of verification experiments (Fig. 4A). In this part of the experiment, in addition to lentivirus, we introduced an additional STING agonist, DMXAA [24, 30, 31]. The results showed that DMXAA inhibited the differentiation and proliferation of osteoclasts in a concentration-dependent manner (Fig. 4B). This inhibitory effect commenced at 10 μm and peaked at 30–50 μm (Fig. 4C, D). The mitogen-activated protein kinase (MAPK) signaling pathway is a key regulatory pathway for osteoclast differentiation, and its importance is undeniable [32]. Our results showed that DMXAA inhibited the activation of the MAPK signaling pathway in a time-dependent manner, further confirming that at the osteoclast level, STING activation could almost certainly inhibit the differentiation of macrophages into osteoclasts (Fig. 4E).

Shifting our focus to tumor cells, we continued our research with MDA-MB-231 and 4T1, two classic breast cancer cell lines. We first determined that increasing DMXAA concentration inhibited the viability of tumor cells, just as it inhibited osteoclast differentiation (Fig. 4F, G). It exerted a similar effect on osteoclast activity (Supplementary Fig. 2A). Subsequently, for a more precise investigation of the regulatory role of STING in the vicious cycle between osteoclasts and breast cancer cells, we overexpressed STING intracellularly using genetic tools and conducted protein-level validation (Fig. 4H). IF experiments with a green fluorescent protein (GFP) demonstrated successful intracellular viral infection (Supplementary Fig. 2B). Moreover, the inherently powerful self-renewal and proliferation potential of tumor cells was effectively suppressed under the premise of overexpression (Fig. 4I).

To simulate the vicious cycle between breast cancer bone metastasis and the formation of a premetastatic niche by osteoclasts, we co-cultured breast cancer cells with osteoclasts and subjected the upper layer of the tumor cells to various stimuli to observe their effects on osteoclast differentiation and proliferation (Fig. 4J). Through the co-cultivation of human breast cancer cells with pre-osteoclasts, we observed that while osteoclast differentiation induced by RANKL was robust, pre-osteoclasts co-cultivated with breast cancer cells exhibited an accelerated rate of differentiation into osteoclasts. Interestingly, this enhanced osteoclast differentiation capability was effectively rescued by stimulating the upper layer of tumor cells with the STING agonist DMXAA (Fig. 4K). Consistent with osteoclast differentiation, the proliferation of pre-osteoclasts was suppressed by DMXAA (Fig. 4L). When the upper layer of breast cancer cells was replaced with murine-derived cells, the differentiation and proliferation of osteoclasts and the rescue effect of DMXAA on this process were consistent with those observed in human-derived cells (Fig. 4M, N). Following infection with lentivirus rather than pharmacological treatment, which allowed for more precise genetic regulation, the conclusions were further corroborated by the increased reliability (Fig. 4O–R).

Finally, we changed the positions of tumor cells and osteoclasts in the co-culture system and treated the upper osteoclasts to observe their effects on tumor cells (Fig. 4S). 5-ethynyl-2′-deoxyuridine (EdU) labeling of the lower-layer tumor cells revealed that the differentiation of the upper-layer osteoclasts significantly enhanced the proliferative activity of the lower-layer tumor cells. However, this amplifying effect on tumor cell viability was abolished when the upper layer of pre-osteoclasts was infected with lentiviral vectors overexpressing STING (Fig. 4T, U). Collectively, through the above experiments at the cellular level, STING has been proven to effectively intervene in the cycle between osteoclast differentiation, tumor proliferation, and metastasis.

### Regulation of the vicious cycle of breast cancer bone metastasis by STING depends on IL-6/STAT3

IL-6 is an inflammatory cytokine associated with chromosomal instability (CIN). CIN leads to the release of micronuclei into the cytoplasm, activating inflammatory signaling mediated by cGAS and STING [33]. IL-6 binds to its receptor complex, IL-6R/gp130, activating downstream Janus kinases (JAKs), which in turn phosphorylate and activate signal transducers and activators of transcription 3 (STAT3). Ultimately, the interaction between these components drives tumor metastasis [9].

Therefore, we understand that research targeting the OPN involving STING must consider the IL-6/STAT3 pathway. As expected, CM derived from tumor cells effectively upregulated intraosseous IL-6 factors and their downstream activated phosphorylated STAT3 levels upon intraperitoneal injection. Notably, this upregulation of activated proteins was absent in the CM derived from STING-expressing cells (Supplementary Fig. 3A–D), suggesting the involvement of the IL-6/STAT3 pathway in regulating OPN formation. Subsequently, to further validate the expression of the STING–IL-6–STAT3 axis in both primary breast cancer tissues and those following bone metastasis, we collected tissue sections from clinical patients who underwent surgical treatment. Consistent with the observations in mouse tissues, metastatic tumor tissues exhibited weakened STING activation along with strong upregulation of downstream IL-6 and STAT3 signaling (Supplementary Fig. 4A, D). This was accompanied by enhanced tumor cell viability (Supplementary Fig. 4B, D) and robust co-expression of bone-specific markers CTSK and Netrin-1 (Supplementary Fig. 4C, D).

**Fig. 5:**
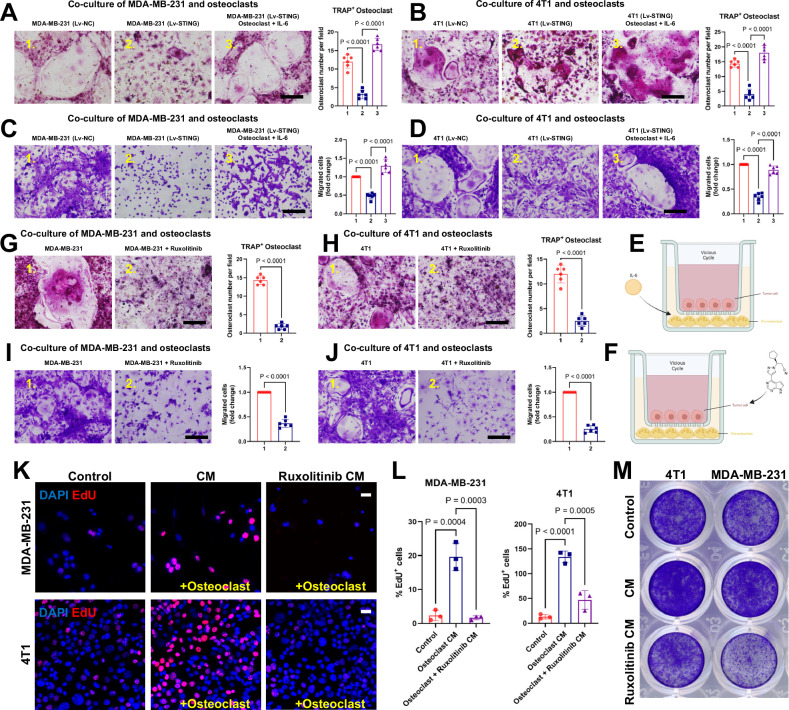
Regulation of the vicious cycle of breast cancer bone metastasis by STING depends on IL-6/STAT3. **A**–**D** Representative images of cells from the migration assay (stained with crystal violet) (bottom) and osteoclast differentiation assay (stained with TRAP) (top), accompanied by corresponding quantitative analysis. The upper layer of tumor cells was either activated for STING via DMXAA treatment or pre-infected with lentivirus expressing STING. IL-6 was added to the lower layer of osteoclasts in the third column. Scale bars, 0.1 cm. *n* = 6 experiments. **E** Model of co-culture of breast cancer cells with preosteoclasts. Murine BMDM preosteoclasts were seeded into the lower chamber plates. Breast cancer cells were seeded into the cell culture inserts in the upper chamber plates and treated with DMXAA or lentivirus expressing STING. Additionally, IL-6 was added to the bottom chamber plates according to experimental requirements. **F** Model of co-culture of breast cancer cells with preosteoclasts, differing from (**E**) by incorporating ruxolitinib into the upper chamber inserts according to experimental design. **G**–**J** Representative images of cells from the migration assay (stained with crystal violet) (bottom) and osteoclast differentiation assay (stained with TRAP) (top), accompanied by corresponding quantitative analysis. The upper layer of tumor cells was supplemented with IL-6/STAT3 inhibitor ruxolitinib as required by the experiment. Scale bars, 0.1 cm. *n* = 6 experiments. **K**, **L** EdU labeling analysis of the impact of osteoclast differentiation on the proliferation of tumor cells, accompanied by corresponding quantitative analysis (**L**). Osteoclasts were cultured in CM with or without the addition of ruxolitinib. Scale bars, 25 µm. *n* = 3 per group. **M** Representative images of cancer cells from the migration assay, stained with crystal violet. Data are presented as mean ± SD. Significance levels are indicated on top of each comparison. Comparisons between the two groups in (**G**–**J**) were performed using an unpaired two-sided Student’s *t*-test. *P* values in (**A**–**D**) and **L** were calculated using a one-way ANOVA with a multiple comparisons test.

To verify this hypothesis more intuitively, we conducted a series of cell-level verifications. Consistent with the methods outlined in Fig. 4, tumor cells were initially positioned in the upper layer of the co-culture system to determine whether the differentiation ability of the lower-layer osteoclasts regulated by STING could be modulated by IL-6 (Fig. 5E). In this experimental group, both the human-derived tumor cell line MDA-MB-231 and murine-derived 4T1 cells infected with viruses expressing STING inhibited the differentiation of the lower-layer osteoclasts significantly compared to the control group. However, this intervention on osteoclast differentiation was ineffective when IL-6 was simultaneously replenished (Fig. 5A–D). As a classical inhibitor of JAK, ruxolitinib not only represses IL6/STAT3 activity and in vivo invasion but also inhibits the release of senescence-associated secretory phenotype (SASP) molecules [9, 34]. Therefore, if it can intervene in the vicious cycle of breast cancer cells and promote osteoclast differentiation, it will further enhance IL-6/STAT3 involvement in this critical process (Fig. 5F). The results were equally promising, as the addition of ruxolitinib directly inhibited the activation of tumor cells and promoted osteoclast differentiation and proliferation, directly illustrating the significance of IL-6/STAT3 in this process (Fig. 5G–J). When the positions of tumor cells and osteoclasts were reversed, EdU staining indicated suppression of proliferation of the lower layer of tumor cells by inhibiting IL-6/STAT3 (Fig. 5K, L). Additionally, results from crystal violet staining further suggested that CM derived from osteoclasts no longer exhibited a positive promoting effect on tumor cell proliferation when intracellular IL-6/STAT3 activation was concurrently suppressed (Fig. 5M). In summary, these results confirm the involvement of IL-6/STAT3 in the vicious cycle regulated by STING. IL-6 supplementation abolished the inhibitory effect of STING on osteoclast differentiation. Moreover, interventions targeting IL-6 and STAT3 can effectively ameliorate the reciprocal promotion of tumor cell proliferation and osteoclast differentiation.

### Hijacking of STING expression in tumor cells by IL-6/STAT3 inhibits OPN formation and bone destruction

In vivo experiments were conducted to investigate the role of IL-6/STAT3 in the vicious cycle of bone metastasis and osteoclast differentiation regulated by STING. Before implanting tumor cells into mice, we artificially created a premetastatic niche for osteoclast tumor metastasis by continuously injecting CM. Subsequently, we intracardially or intravenously injected mice with either the empty vector or an STING-overexpressing virus. A subset of the STING-overexpressing virus group was randomly selected for intraperitoneal injection of IL-6. Through in vivo live animal imaging experiments, we found that effective expression of STING rescued tumor growth and distant metastasis promoted by OPN formation. However, this rescue effect was significantly compromised when co-injected with IL-6 (Supplementary Fig. 5). X-ray imaging data further supported this conclusion. Compared to the intact bone observed in the STING-activated group, the group pre-injected with CM exhibited significant bone metastasis and destruction in common tumor-burden areas.

**Fig. 6:**
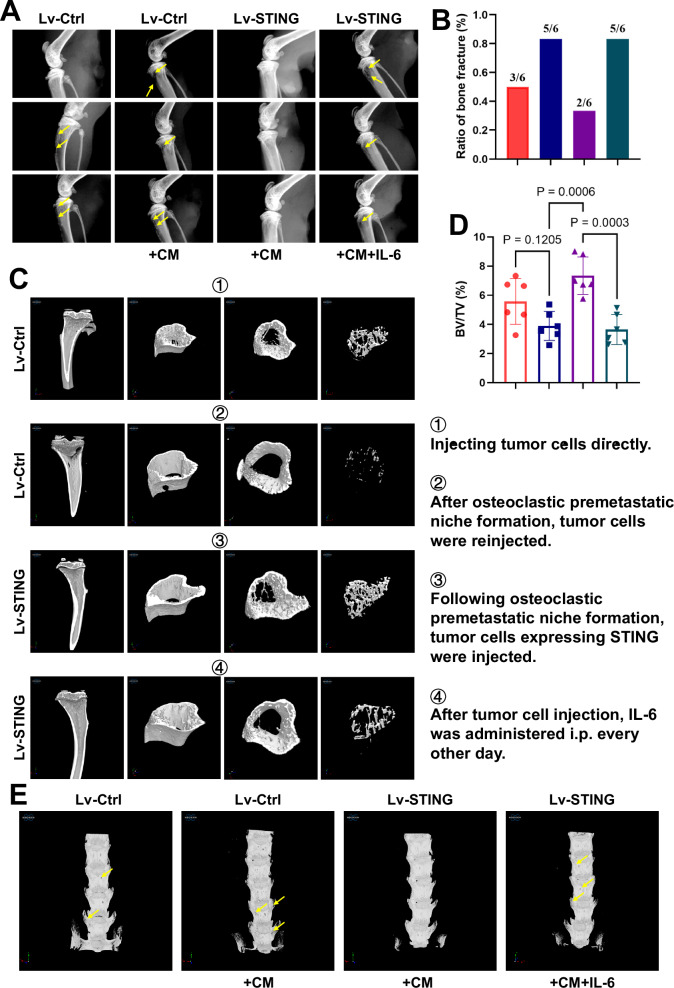
The regulatory role of STING in tumor-induced bone destruction via OPN formation relies on the inhibition of IL-6. **A** Representative radiographs of long bones hosting tumors from mice treated with vehicle or CM, where CM was derived from tumor cells infected with lentivirus expressing STING as needed. The yellow arrows indicate sites of bone destruction. **B** Ratio of bone fractures on day 21 after injection of cancer cells. n = 6 per group. **C** Micro-CT images showing trabecular and cortical bone destruction in the proximal tibia. **D** Morphometric quantification of cortical bone micro-CT images with analysis of BV/TV from different groups. *n* = 6 per group. **E** Micro-CT images showing bone destruction on the surface of the vertebral bodies. The yellow arrows indicate sites of bone destruction. Data are presented as mean ± SD. Significance levels are indicated on top of each comparison. *P* values were calculated using a two-way ANOVA with a multiple comparisons test.

**Fig. 7:**
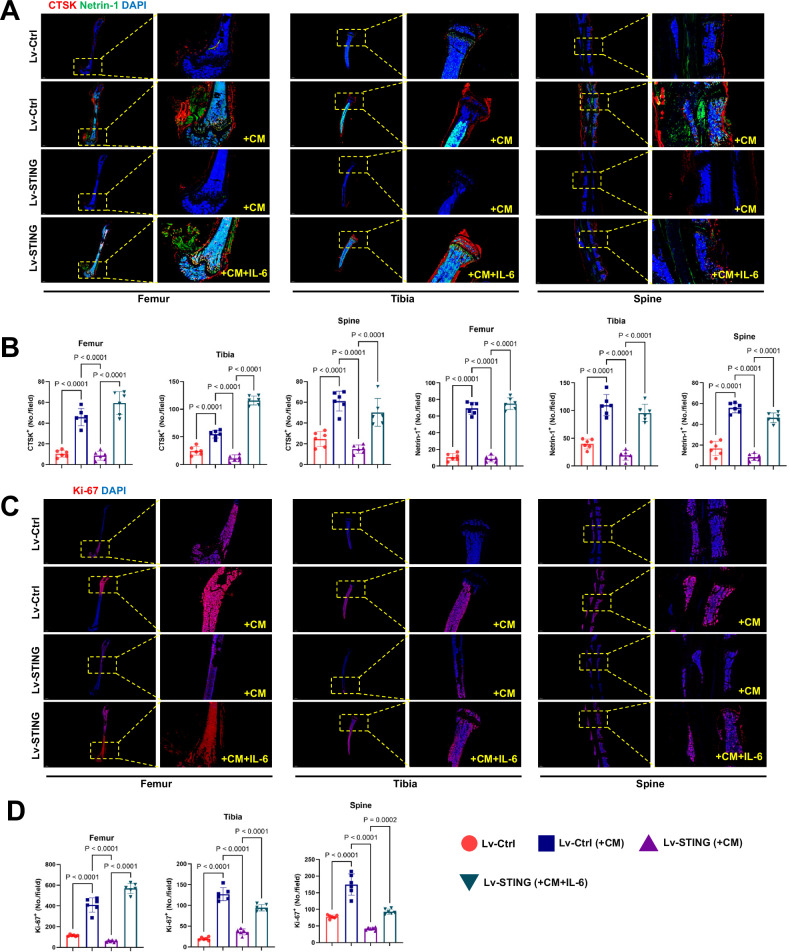
Hijacking of STING expression in tumor cells by IL-6/STAT3 inhibits OPN formation and bone destruction. **A**, **B** Representative images of IF staining of CTSK (red) and Netrin-1 (green) in the femur, tibia, and spine (top) and quantitative analysis (bottom). Scale bars, 1000 µm. *n* = 6 per group. **C**, **D** Representative immunofluorescent images (**C**) and quantification (**D**) of proliferative tumor cells in the femur, tibia, and spine (*n* = 6 mice), as determined by the presence of Ki-67^+^. Scale bars, 1000 µm. Data are presented as mean ± SD. Significance levels are indicated on top of each comparison. *P* values were calculated using a two-way ANOVA with a multiple comparisons test.

Additionally, in the IL-6 group, continuous injection of IL-6 weakened the protective effect of STING (Fig. 6A, B). Micro-CT scanning and three-dimensional reconstruction provided more intuitive perspectives. Premetastatic niche formation significantly reduces the thickness of the bone cortex, leading to bone destruction and further disruption of the bone marrow content. Overexpression of STING inhibited the formation of a premetastatic niche, suppressing premature bone metastasis and destruction. However, this protective effect was not observed in the presence of IL-6 (Fig. 6C). Consistent changes were observed in the bone volume fraction (BV/TV), representing bone mass (Fig. 6D). Shifting our focus to another common site of bone metastasis, the spine, we observed that the trend of tumor metastasis and destruction on the surface of the spine was consistent with that of the long bones (Fig. 6E). Double staining for CTSK and Netrin-1 further demonstrated that the enhanced effect of OPN formation rescued by STING on bone destruction was reversed under conditions of IL-6 overexpression at bone metastatic sites in both models (Fig. 7A, B). Moreover, although we did not directly label tumor cells within the bone, cells exhibiting robust proliferative activity, as indicated by Ki-67 staining, showed strong expression in the group promoted by the OPN. This strong expression suppressed by STING was partially restored by IL-6 (Fig. 7C, D). In summary, through both in vivo and in vitro experiments, we have demonstrated that the disruption of the vicious cycle between OPN formation, regulated by STING, and tumor bone metastasis relies on its inhibition of IL-6/STAT3. This finding emphasizes the importance of addressing this issue in clinical translation.

## Discussion

Metastasis of tumor cells to the bone presents a clinically significant challenge. In recent years, there has been a surge in fundamental scientific research addressing this issue. While most researchers in the field of tumor-bone metastasis recognize the pivotal role of osteoclasts in this process, limited attention has been paid to the premetastatic niche that osteoclasts form before substantial tumor-driven bone metastasis occurs. This premetastatic microenvironment, orchestrated by osteoclasts, plays a crucial guiding and facilitative role in tumor metastasis, a dimension largely overlooked by existing research efforts. In this study, we observed that the formation of an OPN significantly promotes the metastasis and dissemination of non-osseous-origin tumors to the bone.

Moreover, we found that the effective expression of STING mitigated this pro-metastatic effect. Furthermore, the vicious cycle formed by the attractive effect of the premetastatic microenvironment, resulting from excessive osteoclast differentiation on tumor cell proliferation and metastasis, along with the tumor cell-promoting effect of osteoclast differentiation, requires IL-6 and STAT3. Highly activated IL-6/STAT3 abolishes the protective effects of STING on bone metastasis. These results reveal the crucial interplay between STING-regulated IL-6/STAT3 in the formation of the OPN and tumor bone metastasis, providing compelling evidence and clinical intervention targets.

Most theories regarding osteoclasts in tumor bone metastasis consider them merely as a passive “terminal station,” focusing on their role in bone destruction following substantial tumor metastasis, often overlooking their potential attraction towards primary bone tumors [15, 24, 35–37]. This study posits that before tumor metastasis, osteoclast differentiation within the bone microenvironment becomes active, indicating the direction of tumor migration. Its role resembles more that of a “lighthouse on the open sea.” Previous studies inspire this study. As early as 2022, studies emphasized the importance of the pre-metastatic niche created by osteoclasts in bone metastasis and identified RSPO2/RANKL-LGR4 signaling as a promising target for inhibiting bone metastasis [14]. Yunfei He et al.‘s research highlighted that osteoclasts are not merely “bone digesters”; they provide physical space for tumor growth and release protumor factors from degraded bone matrix, facilitating tumor colonization in bone, which is equally deserving of attention [11]. Additionally, the influence of inflammatory cytokines and chemokines from within the bone on the entire body also plays a crucial guiding role in tumor metastasis to the bone [8, 10, 11, 38]. In addition to the factors mentioned above, we believe that the formation of the pre-metastatic niche requires attention to factors more closely related to osteoclasts, such as CTSK and Netrin-1, as highlighted in this study. Wang et al. recently pointed out that the factor LTβ promotes the colonization and growth of breast cancer cells in various models [39]. Further mechanistic studies found that tumor-derived LTβ activates osteoblasts through the NF-κB2 signaling pathway, leading to the secretion of CCL2/5, which in turn facilitates the adhesion of tumor cells to osteoblasts and accelerates osteoclastogenesis, thereby driving the progression of bone metastasis [39].

Cathepsin K (CTSK) is a novel cysteine protease previously reported to be predominantly expressed in osteoclasts [40]. Immunolocalization studies on breast tumor bone metastases revealed the expression of this protease in invasive breast cancer cells, albeit at a lower intensity than in osteoclasts, suggesting a potential role of CTSK in the invasiveness of breast cancer cells [35, 41, 42]. CTSK promotes bone resorption, creating a favorable bone microenvironment for tumor growth [42], which could be a significant factor in osteoclasts promoting tumor metastasis. In this study, we found that the formation of a premetastatic niche significantly promotes CTSK expression in the bone microenvironment. This suggests that a conducive environment for tumor growth and dissemination may already be established before tumor metastasis occurs. Tumors inherently possess the potential for vascular metastasis, and Netrin-1 has been shown to influence angiogenesis through various pathways [43–46]. As a pro-tumorigenic mechanism, Netrin-1 is upregulated in most human endometrial carcinomas (ECs) [38, 47]. Research has also demonstrated the efficacy of blocking Netrin-1 using an anti-Netrin-1 antibody (NP137) to reduce tumor progression in an EC mouse model [27].

Additionally, Netrin-1 can be secreted by osteoclasts [28]. This study found that both the premetastatic niche formed before substantial tumor dissemination and the intraosseous microenvironment established after metastasis displayed prominent fluorescence staining for CTSK and Netrin-1. Therefore, we speculate that targeting these two proteins alone may counteract the beneficial effects of the pre-metastatic niche on tumor bone metastasis.

In recent years, manipulation of the cGAS–STING pathway has garnered significant interest in tumor immunology. STING agonizts alone or in combination with ICB therapy are currently under clinical investigation for their potential as a new class of anticancer treatment [48]. Systemic delivery of STING agonizts induces IFN -independent cytopathic effects in tumor cells [49, 50]. Recent studies have confirmed the inhibitory effects of STING agonizts on tumor-induced bone destruction and cancer-related skeletal pain [24, 51]. While direct tumor cell-mediated bone destruction within the bone has been reported, the ability of STING to intervene in tumor-to-bone colonization by modulating the relevant microenvironment formed by osteoclasts before the tumor metastasizes to the bone remains unclear. Indeed, STING-dependent cell death responses may also suppress tumor clearance. Tumor transplantation studies have demonstrated that cancer cells can effectively utilize cGAS-STING signaling to kill T cells, likely through cGAMP secretion [52, 53].

Similarly, endothelial cells tend to undergo apoptosis after exposure to cGAS-STING triggering factors [54]. Various cell type-specific immunomodulatory responses have been documented, which could inhibit the tumor-suppressive effects of STING activation [55]. Therefore, the overall effect of STING on tumor progression vs regression is highly dependent on the context, and investigating the microenvironment preceding osteoclast migration holds significant research value. Our findings suggest that both the specific STING-targeting agonist DMXAA and STING-expressing lentivirus infection can effectively inhibit the formation of a premetastatic niche and suppress tumor-induced bone destruction in this niche, highlighting the protective role of STING in OPN formation and promotion of tumor metastasis in this context.

Overexpression of IL-6 in the tumor microenvironment is closely associated with tumor development, infiltration, and metastasis, thus making it a potential therapeutic target for tumor progression [33, 56]. IL-6 binds to its receptor complex IL6R/gp130, activating downstream JAKs that subsequently phosphorylate tyrosine 705 of STAT3 [57]. The expression levels of pSTAT3 have been confirmed to be closely associated with breast cancer prognosis, particularly in patients with IL6-positive tumors [58–60]. Therefore, assessing both pSTAT3 and IL6 positivity is crucial for understanding the clinical impact of activating this pathway in patients.

While our study primarily focuses on breast cancer bone metastasis, the mechanisms elucidated here—particularly the formation of an OPN regulated by the STING-IL-6/STAT3 axis—may have broader relevance to other tumor types that metastasize to bone, such as prostate cancer, lung cancer, and multiple myeloma. These cancers share common features in their interactions with the bone microenvironment, including osteoclast activation and niche formation, suggesting potential applicability of our findings beyond breast cancer. Clinically, targeting the STING pathway and key niche components like CTSK and Netrin-1 offers promising avenues for novel therapeutic interventions aimed at preventing or mitigating bone metastases. Future research should investigate the role of this premetastatic niche and STING signaling in other tumor contexts, explore combinational therapies that incorporate STING agonizts with existing treatments, and delineate the temporal dynamics of niche formation and tumor colonization. Additionally, key questions remain regarding the cell type-specific effects of STING activation and the balance between its tumor-suppressive and tumor-promoting functions in different microenvironments. Addressing these issues will be critical for translating our findings into effective clinical strategies to improve outcomes for patients suffering from bone metastatic disease.

## Conclusion

In summary, our study demonstrated that sole inhibition of IL-6/STAT3 effectively disrupted the vicious cycle of bone metastasis. Moreover, STING’s regulation of premetastatic niche formation is dependent on IL-6 and STAT3. Given the various factors involved in tumorigenesis that can upregulate IL-6 levels [61, 62], identifying this mechanism is paramount.

## Materials and methods

### Mice

Female BALB/C mice (6–8-weeks-old) were obtained from Gempharmatech and had ad libitum access to drinking water. Mice were maintained in a pathogen-free environment. Primary osteoclasts were isolated from 6- to 8-week-old BALB/C mice. All experiments involving mice were performed in accordance with the guidelines of the Institutional Research Ethics Committee of the Shanghai Ninth People’s Hospital (SH9H-2024-A995-1).

### Human cancer samples

Written informed consent was obtained from all participants prior to their inclusion in the study. The research was conducted in accordance with the ethical standards of the Institutional Research Ethics Committee of the Shanghai Ninth People’s Hospital. Primary breast cancer tissues and bone metastatic lesions were obtained from female patients diagnosed with breast cancer who underwent surgical resection at, following informed consent and approval by the Institutional Ethics Committee. Inclusion criteria included histologically confirmed primary breast carcinoma or bone metastatic lesions, availability of sufficient tissue for analysis, and no prior neoadjuvant chemotherapy. Tissue specimens were collected intraoperatively by experienced pathologists and immediately fixed in 10% neutral-buffered formalin for 24–48 h, followed by paraffin embedding. Serial sections were prepared for immunohistochemical and IF analyses. All specimens were anonymized prior to analysis to ensure patient confidentiality.

### Cell culture

MDA-MB-231 (human breast cancer) and 4T1 (mouse breast cancer) cells were acquired from Pricella and cultured in DMEM (Gibco) supplemented with 10% fetal bovine serum (FBS).

For bone marrow-derived macrophages (BMDMs), 6–8-week-old mice were used. After euthanasia and dissection of the hind limbs, femurs and tibias were harvested. The bone epiphyses at both ends were removed, and the bone marrow was flushed out using a 1 mL insulin syringe. The harvested cells were cultured either in a 10 cm dish or transferred to a T75 flask. The culture medium comprised a complete medium containing 30 ng/mL M-CSF, 10% FBS, and penicillin-streptomycin. After two days, the cells were observed under a microscope, and the medium was changed to a fresh medium (α-MEM was chosen as the culture medium). After an additional two days, numerous mononuclear macrophages derived from the bone marrow were visible under the microscope, indicating successful extraction of BMDMs. For further induction of their differentiation into osteoclasts, BMDMs were trypsin-digested, plated, and after cell adhesion, cultured for 5–6 consecutive days in α-MEM complete medium containing 30 ng/mL M-CSF and 100 ng/mL RANKL. The observation of a significant number of ginkgo leaf-like fused cells indicated the initiation of osteoclast fusion.

### Generation of STING-expressing cells

MDA-MB-231, 4T1, and pre-osteoclasts were seeded in a 6-well plate at a density of 2 × 10^5^ cells per well and cultured overnight. Lentiviral particles carrying either the empty vector or the STING gene were obtained from OBiO Technology (Shanghai, China). The lentiviral vectors were added to the cells along with polybrene (8 μg/mL) to enhance transduction efficiency. After 24 h of transduction, the medium was replaced with a fresh growth medium. Transduced cells were selected using appropriate antibiotics or fluorescent markers, and overexpression of the target gene was confirmed by IF and western blot analyses.

### CM collection

The CM was collected and referenced following established protocols [14, 63, 64]. A total of 1 × 10^6^ 4T1 and 2 × 10^6^ MDA-MB-231 cells, infected with either an empty viral vector or STING-expressing virus, were plated in a 10 cm dish and cultured until they reached 85% confluence. For DMXAA treatment, cells were stimulated with 10 μM DMXAA (HY-10964, MCE). Subsequently, the cells were washed twice with phosphate-buffered saline (PBS) and cultured for 48 h in serum-free DMEM. The CM was then collected, filtered through a 0.22 μm filter to remove cell debris, and concentrated using a Millipore protein concentrator. The medium was centrifuged at 3500×*g* until a triple concentration was achieved. The concentrated CM was aliquoted to prevent repeated freezing and thawing and stored at −80 °C for use within 3 months.

For the premetastatic niche mouse model, 300 μL of CM or control medium (serum-free DMEM) was intraperitoneally injected daily into 6-week-old BALB/C mice for 3 weeks or as indicated to induce premetastatic niche formation.

### Histology and IF

Mouse bones were fixed in 4% paraformaldehyde, decalcified, dehydrated, and embedded in paraffin. TRAP staining was utilized to assess the extent of osteoclast differentiation in the long bones, spine, and skull. For IF staining, tissue sections were permeabilized with 0.5% Triton X-100, blocked with 5% BSA for 1 h, and then incubated with primary antibodies overnight at 4 °C. Subsequently, sections were washed three times and incubated with secondary antibodies (goat anti-rabbit IgG H&L [Alexa Fluor® 555; Abcam] or goat anti-mouse IgG H&L [Alexa Fluor® 488; Abcam], diluted 1:200) for 1 h the following day. Quantitative IF staining was performed in a double-blind manner. The primary antibodies used in this study were Cathepsin K Polyclonal antibody (Proteintech, cat. # 11239-1-AP), Rabbit monoclonal to Netrin-1 (Abcam, cat. # ab126729), and rabbit anti-mouse Ki-67 (Abcam, cat. # ab15580).

### Radiographic analysis

Digital X-ray imaging of the long bones and spine was conducted according to the manufacturer’s guidelines [65], utilizing a 21 lp/mm detector in the anteroposterior axis, with up to 5× geometric magnification (Faxitron VersaVision; Faxitron Bioptics LLC, Tucson, AZ, USA).

### Micro-computed tomography (micro-CT)

Mice's bones were dissected to remove soft tissue, then fixed overnight in 70% ethanol and analyzed using high-resolution micro-CT (μCT) (Skyscan1275, Bruker micro-CT, Kontich, Belgium). The three-dimensional model visualization software CTVol was utilized for the analysis of trabecular bone parameters in the epiphysis, as well as for an overall reconstruction.

### Cell counting kit-8 (CCK-8) cell proliferation assay

For the cell proliferation assay, cells were seeded in 96-well plates at a density of 1 × 10^4^ cells/well and incubated for 24 h. Subsequently, the cells were treated with different concentrations of chemical reagents. Cell proliferation was assessed at the designated endpoint using a CCK-8 assay (C0037; Beyotime). Absorbance at 450 nm (mean optical density) was measured using an Infinite M200 Pro multimode microplate reader (Tecan Group Ltd., Männedorf, Switzerland).

### ELISA

To assess the expression levels of STING protein in the supernatant of tumor cell cultures, an ELISA (enzyme-linked immunosorbent assay) (ab315058, abcam) kit was used according to the manufacturer’s protocol.

### Cell co-culture

A dual co-culture assay and staining were performed to investigate osteoclast activity. Pre-osteoclasts from murine bone marrow macrophages (1 × 10^5^ cells/well) were directly seeded into 12-well co-culture plates. Tumor cells (5 × 10^4^ cells/well) were seeded into Transwell® cell culture inserts (Costar) placed within the co-culture plates. The following day, the tumor cells adhered to the membrane surface of the inserts, and various stimuli, such as chemical agents or viruses, were added to the top of the culture. The upper co-culture assays were conducted in DMEM/high-glucose medium supplemented with 10% FBS, with media changes every 2 days. The lower chambers were maintained using α-MEM complete medium supplemented with M-CSF (30 ng/mL) and RANKL (100 ng/mL). On day 4, TRAP staining of osteoclasts was performed, and TRAP-positive multinucleated cells were identified and quantified as mature osteoclasts. Interchanging cells between the upper and lower layers followed the method described above.

### Western blot

Cultured cells were lysed with RIPA lysis buffer supplemented with phosphatase and protease inhibitors. Total protein was quantified using the bicinchoninic acid assay (Thermo Fisher Scientific). Equal amounts of extracted protein (20–30 μg) were separated using 12% or 15% sodium dodecyl sulfate-polyacrylamide gel electrophoresis and transferred onto 0.22-μm PVDF membranes (MilliporeSigma, Burlington, MA, USA). Membranes were blocked with 5% BSA-PBS (Beyotime Biotechnology) at room temperature (RT = 25 °C) for 1 h, then incubated overnight with primary antibodies at 4 °C. The next day, membranes were washed with Tris-buffered saline (TBS)-0.1% Tween 20 (TBST) and incubated with an anti-rabbit IgG (H + L) secondary antibody (cat. no. 5151; DyLight™ 800 4X PEG Conjugate; Cell Signaling Technology; 1:10,000) for 1 h at RT in the dark. After washing with TBST, protein immunoreactivity was detected using an Odyssey Fluorescence Imaging system (LI-COR Biosciences, Lincoln, NE, USA). The primary antibodies used in this study were as follows: Rabbit monoclonal to STING (Cell Signaling Technology, cat. # 13647); MAPK Family Antibody Sampler Kit (Cell Signaling Technology, cat. # 9926).

### EdU labeling

Cells were cultured with 10 μM EdU for 24 h. Following incubation, the cells were stained using the Click Plus EdU 647 Imaging Kit (Thermo Fisher, C10640). This kit facilitates visualization and detection of EdU incorporated into cellular DNA, providing insights into cell proliferation dynamics and activity.

### Bioluminescent

For bioluminescent imaging, mice were anesthetized and retro-orbitally injected with 1.5 mg D-luciferin at specified time points. Imaging was performed in an IVIS 100 chamber within 5 min following D-luciferin injection, with data acquisition conducted using Living Image software (Xenogen).

### Pharmacological compounds

DMXAA (HY-10964, MCE) and Ruxolitinib (HY-50856) were procured from MCE (MedChemExpress). All pharmacological compounds were prepared according to the manufacturer’s instructions and stored under the appropriate conditions.

### Statistical analysis

Statistical analysis of all data was conducted using GraphPad Prism software (version 8.0). Results are presented as the mean ± SD. Comparisons between the two groups were performed using an unpaired two-sided Student’s *t*-test (*P* < 0.05 was deemed significant). One- or two-way analysis of variance was used for comparisons between multiple conditions. Specific *n* values are provided in the figure legends. In the graphs, the individual data points are depicted as dots. No statistical method was used to predetermine the sample size. Instead, sample sizes were determined based on historical sample sizes capable of detecting biologically significant differences in certain assays, with p < 0.05 considered statistically significant.

## Supplementary information

Figures S1–S5

The full uncropped Gels and Blots image(s)

## Data Availability

The datasets supporting the conclusions of this article are included within the article and its additional files.

## References

[1] Fornetti J, Welm AL, Stewart SA. Understanding the bone in cancer metastasis. J Bone Min Res. 2018;33:2099–113. 10.1002/jbmr.3618 30476357

[2] Satcher RL, Zhang XH. Evolving cancer-niche interactions and therapeutic targets during bone metastasis. Nat Rev Cancer. 2022;22:85–101. 34611349 10.1038/s41568-021-00406-5 PMC10281546

[3] Weilbaecher KN, Guise TA, McCauley LK. Cancer to bone: a fatal attraction. Nat Rev Cancer. 2011;11:411–25. 21593787 10.1038/nrc3055 PMC3666847

[4] Zhang W, Bado IL, Hu J, Wan YW, Wu L, Wang H, et al. The bone microenvironment invigorates metastatic seeds for further dissemination. Cell. 2021;184:2471–86.e2420. 33878291 10.1016/j.cell.2021.03.011 PMC8087656

[5] Guise TA, Yin JJ, Taylor SD, Kumagai Y, Dallas M, Boyce BF, et al. Evidence for a causal role of parathyroid hormone-related protein in the pathogenesis of human breast cancer-mediated osteolysis. J Clin Invest. 1996;98:1544–9. 8833902 10.1172/JCI118947 PMC507586

[6] Kang Y, Siegel PM, Shu W, Drobnjak M, Kakonen SM, Cordón-Cardo C, et al. A multigenic program mediating breast cancer metastasis to bone. Cancer Cell. 2003;3:537–49. 12842083 10.1016/s1535-6108(03)00132-6

[7] Parida PK, Marquez-Palencia M, Ghosh S, Khandelwal N, Kim K, Nair V, et al. Limiting mitochondrial plasticity by targeting DRP1 induces metabolic reprogramming and reduces breast cancer brain metastases. Nat Cancer. 2023;4:893–907. 37248394 10.1038/s43018-023-00563-6 PMC11290463

[8] Romero-Moreno R, Curtis KJ, Coughlin TR, Miranda-Vergara MC, Dutta S, Natarajan A, et al. The CXCL5/CXCR2 axis is sufficient to promote breast cancer colonization during bone metastasis. Nat Commun. 2019;10:4404. 31562303 10.1038/s41467-019-12108-6 PMC6765048

[9] Siersbaek R, Scabia V, Nagarajan S, Chernukhin I, Papachristou EK, Broome R, et al. IL6/STAT3 signaling hijacks estrogen receptor alpha enhancers to drive breast cancer metastasis. Cancer Cell. 2020;38:412–23.e419. 32679107 10.1016/j.ccell.2020.06.007 PMC7116707

[10] Su W, Han HH, Wang Y, Zhang B, Zhou B, Cheng Y, et al. The polycomb repressor complex 1 drives double-negative prostate cancer metastasis by coordinating stemness and immune suppression. Cancer Cell. 2019;36:139–55.e110. 31327655 10.1016/j.ccell.2019.06.009 PMC7210785

[11] He Y, Luo W, Liu Y, Wang Y, Ma C, Wu Q, et al. IL-20RB mediates tumoral response to osteoclastic niches and promotes bone metastasis of lung cancer. J Clin Invest. 2022;132:e157917. 36006737 10.1172/JCI157917 PMC9566910

[12] Seo J, Kim H, Min KI, Kim C, Kwon Y, Zheng Z, et al. Weight-bearing activity impairs nuclear membrane and genome integrity via YAP activation in plantar melanoma. Nat Commun. 2022;13:2214. 35468978 10.1038/s41467-022-29925-x PMC9038926

[13] Zhang L, Qu J, Qi Y, Duan Y, Huang YW, Zhou Z, et al. EZH2 engages TGFbeta signaling to promote breast cancer bone metastasis via integrin beta1-FAK activation. Nat Commun. 2022;13:2543. 35538070 10.1038/s41467-022-30105-0 PMC9091212

[14] Yue Z, Niu X, Yuan Z, Qin Q, Jiang W, He L, et al. RSPO2 and RANKL signal through LGR4 to regulate osteoclastic premetastatic niche formation and bone metastasis. J Clin Invest. 2022;132:e144579. 34847079 10.1172/JCI144579 PMC8759794

[15] Wang K, Gu Y, Liao Y, Bang S, Donnelly CR, Chen O, et al. PD-1 blockade inhibits osteoclast formation and murine bone cancer pain. J Clin Invest. 2020;130:3603–20. 32484460 10.1172/JCI133334 PMC7324182

[16] Liu Y, Cao X. Characteristics and significance of the pre-metastatic niche. Cancer Cell. 2016;30:668–81. 27846389 10.1016/j.ccell.2016.09.011

[17] Chen T, Feng Y, Sun W, Zhao G, Wu H, Cheng X, et al. The nucleotide receptor STING translocates to the phagosomes to negatively regulate anti-fungal immunity. Immunity. 2023;56:1727–42.e6. 37379835 10.1016/j.immuni.2023.06.002

[18] Ishikawa H, Barber GN. STING is an endoplasmic reticulum adaptor that facilitates innate immune signalling. Nature. 2008;455:674–8. 18724357 10.1038/nature07317 PMC2804933

[19] Ergun SL, Fernandez D, Weiss TM, Li L. STING polymer structure reveals mechanisms for activation, hyperactivation, and inhibition. Cell. 2019;178:290–301.e210. 31230712 10.1016/j.cell.2019.05.036

[20] Dobbs N, Burnaevskiy N, Chen D, Gonugunta VK, Alto NM, Yan N. STING activation by translocation from the ER is associated with infection and autoinflammatory disease. Cell Host Microbe. 2015;18:157–68. 26235147 10.1016/j.chom.2015.07.001 PMC4537353

[21] Coelho LF, Magno de Freitas Almeida G, Mennechet FJ, Blangy A, Uzé G. Interferon-alpha and -beta differentially regulate osteoclastogenesis: role of differential induction of chemokine CXCL11 expression. Proc Natl Acad Sci USA. 2005;102:11917–22. 16081539 10.1073/pnas.0502188102 PMC1187968

[22] Baum R, Sharma S, Organ JM, Jakobs C, Hornung V, Burr DB, et al. STING contributes to abnormal bone formation induced by deficiency of DNase II in mice. Arthritis Rheumatol. 2017;69:460–71. 27740718 10.1002/art.39863 PMC5274601

[23] Kwon Y, Park OJ, Kim J, Cho JH, Yun CH, Han SH. Cyclic dinucleotides inhibit osteoclast differentiation through STING-mediated interferon-β signaling. J Bone Min Res. 2019;34:1366–75. 10.1002/jbmr.3701 30779854

[24] Wang K, Donnelly CR, Jiang C, Liao Y, Luo X, Tao X, et al. STING suppresses bone cancer pain via immune and neuronal modulation. Nat Commun. 2021;12:4558. 34315904 10.1038/s41467-021-24867-2 PMC8316360

[25] Mehlen P, Delloye-Bourgeois C, Chédotal A. Novel roles for slits and netrins: Axon guidance cues as anticancer targets?. Nat Rev Cancer. 2011;11:188–97. 21326323 10.1038/nrc3005

[26] Sung PJ, Rama N, Imbach J, Fiore S, Ducarouge B, Neves D, et al. Cancer-associated fibroblasts produce Netrin-1 to control cancer cell plasticity. Cancer Res. 2019;79:3651–61. 31088838 10.1158/0008-5472.CAN-18-2952

[27] Cassier PA, Navaridas R, Bellina M, Rama N, Ducarouge B, Hernandez-Vargas H, et al. Netrin-1 blockade inhibits tumour growth and EMT features in endometrial cancer. Nature. 2023;620:409–16. 37532934 10.1038/s41586-023-06367-z PMC10412451

[28] Zhu S, Zhu J, Zhen G, Hu Y, An S, Li Y, et al. Subchondral bone osteoclasts induce sensory innervation and osteoarthritis pain. J Clin Invest. 2019;129:1076–93. 30530994 10.1172/JCI121561 PMC6391093

[29] Barber GN. STING: infection, inflammation and cancer. Nat Rev Immunol. 2015;15:760–70. 26603901 10.1038/nri3921 PMC5004891

[30] Weiss JM, Guérin MV, Regnier F, Renault G, Galy-Fauroux I, Vimeux L, et al. The STING agonist DMXAA triggers a cooperation between T lymphocytes and myeloid cells that leads to tumor regression. Oncoimmunology. 2017;6:e1346765. 29123960 10.1080/2162402X.2017.1346765 PMC5665074

[31] Guerin MV, Regnier F, Feuillet V, Vimeux L, Weiss JM, Bismuth G, et al. TGFβ blocks IFNα/β release and tumor rejection in spontaneous mammary tumors. Nat Commun. 2019;10:4131. 31511510 10.1038/s41467-019-11998-w PMC6739328

[32] Xie X, Cheng P, Hu L, Zhou W, Zhang D, Knoedler S, et al. Bone-targeting engineered small extracellular vesicles carrying anti-miR-6359-CGGGAGC prevent valproic acid-induced bone loss. Signal Transduct Target Ther. 2024;9:24. 38246920 10.1038/s41392-023-01726-8 PMC10800355

[33] Hong C, Schubert M, Tijhuis AE, Requesens M, Roorda M, van den Brink A, et al. cGAS-STING drives the IL-6-dependent survival of chromosomally instable cancers. Nature. 2022;607:366–73. 35705809 10.1038/s41586-022-04847-2

[34] Liu X, Gu Y, Kumar S, Amin S, Guo Q, Wang J, et al. Oxylipin-PPARgamma-initiated adipocyte senescence propagates secondary senescence in the bone marrow. Cell Metab. 2023;35:667–84.e666. 37019080 10.1016/j.cmet.2023.03.005 PMC10127143

[35] Wang S, Li GX, Tan CC, He R, Kang LJ, Lu JT, et al. FOXF2 reprograms breast cancer cells into bone metastasis seeds. Nat Commun. 2019;10:2707. 31222004 10.1038/s41467-019-10379-7 PMC6586905

[36] Sawant A, Deshane J, Jules J, Lee CM, Harris BA, Feng X, et al. Myeloid-derived suppressor cells function as novel osteoclast progenitors enhancing bone loss in breast cancer. Cancer Res. 2013;73:672–82. 23243021 10.1158/0008-5472.CAN-12-2202 PMC3548966

[37] An G, Acharya C, Feng X, Wen K, Zhong M, Zhang L, et al. Osteoclasts promote immune suppressive microenvironment in multiple myeloma: therapeutic implication. Blood. 2016;128:1590–603. 27418644 10.1182/blood-2016-03-707547 PMC5034739

[38] Anstee JE, Feehan KT, Opzoomer JW, Dean I, Muller HP, Bahri M, et al. LYVE-1+ macrophages form a collaborative CCR5-dependent perivascular niche that influences chemotherapy responses in murine breast cancer. Dev Cell. 2023;58:1548–61.e10. 37442140 10.1016/j.devcel.2023.06.006

[39] Wang X, Zhang T, Zheng B, Lu Y, Liang Y, Xu G, et al. Lymphotoxin-beta promotes breast cancer bone metastasis colonization and osteolytic outgrowth. Nat Cell Biol. 2024;26:1597–612. 39147874 10.1038/s41556-024-01478-9

[40] Morko J, Kiviranta R, Mulari MT, Ivaska KK, Väänänen HK, Vuorio E, et al. Overexpression of cathepsin K accelerates the resorption cycle and osteoblast differentiation in vitro. Bone. 2009;44:717–28. 19118660 10.1016/j.bone.2008.11.019

[41] Littlewood-Evans AJ, Bilbe G, Bowler WB, Farley D, Wlodarski B, Kokubo T, et al. The osteoclast-associated protease cathepsin K is expressed in human breast carcinoma. Cancer Res. 1997;57:5386–90. 9393764

[42] Le Gall C, Bellahcène A, Bonnelye E, Gasser JA, Castronovo V, Green J, et al. A cathepsin K inhibitor reduces breast cancer induced osteolysis and skeletal tumor burden. Cancer Res. 2007;67:9894–902. 17942921 10.1158/0008-5472.CAN-06-3940

[43] Park KW, Crouse D, Lee M, Karnik SK, Sorensen LK, Murphy KJ, et al. The axonal attractant Netrin-1 is an angiogenic factor. Proc Natl Acad Sci USA. 2004;101:16210–5. 15520390 10.1073/pnas.0405984101 PMC528958

[44] Wilson BD, Ii M, Park KW, Suli A, Sorensen LK, Larrieu-Lahargue F, et al. Netrins promote developmental and therapeutic angiogenesis. Science. 2006;313:640–4. 16809490 10.1126/science.1124704 PMC2577078

[45] Mehlen P, Furne C. Netrin-1: when a neuronal guidance cue turns out to be a regulator of tumorigenesis. Cell Mol Life Sci. 2005;62:2599–616. 16158190 10.1007/s00018-005-5191-3 PMC11139161

[46] Zheng H, An M, Luo Y, Diao X, Zhong W, Pang M, et al. PDGFRalpha(+)ITGA11(+) fibroblasts foster early-stage cancer lymphovascular invasion and lymphatic metastasis via ITGA11-SELE interplay. Cancer Cell. 2024;42:682–700.e12. 38428409 10.1016/j.ccell.2024.02.002

[47] Esposito M, Mondal N, Greco TM, Wei Y, Spadazzi C, Lin SC, et al. Bone vascular niche E-selectin induces mesenchymal-epithelial transition and Wnt activation in cancer cells to promote bone metastasis. Nat Cell Biol. 2019;21:627–39. 30988423 10.1038/s41556-019-0309-2 PMC6556210

[48] Samson N, Ablasser A. The cGAS–STING pathway and cancer. Nat Cancer. 2022;3:1452–63. 36510011 10.1038/s43018-022-00468-w

[49] Petrasek J, Iracheta-Vellve A, Csak T, Satishchandran A, Kodys K, Kurt-Jones EA, et al. STING-IRF3 pathway links endoplasmic reticulum stress with hepatocyte apoptosis in early alcoholic liver disease. Proc Natl Acad Sci USA. 2013;110:16544–9. 24052526 10.1073/pnas.1308331110 PMC3799324

[50] Curran E, Chen X, Corrales L, Kline DE, Dubensky TW Jr, Duttagupta P, et al. STING pathway activation stimulates potent immunity against acute myeloid leukemia. Cell Rep. 2016;15:2357–66. 27264175 10.1016/j.celrep.2016.05.023 PMC5116809

[51] Donnelly CR, Jiang C, Andriessen AS, Wang K, Wang Z, Ding H, et al. STING controls nociception via type I interferon signalling in sensory neurons. Nature. 2021;591:275–80. 33442058 10.1038/s41586-020-03151-1 PMC7977781

[52] Wu J, Dobbs N, Yang K, Yan N. Interferon-independent activities of mammalian STING mediate antiviral response and tumor immune evasion. Immunity. 2020;53:115–.e115. 32640258 10.1016/j.immuni.2020.06.009 PMC7365768

[53] Concepcion AR, Wagner LE 2nd, Zhu J, Tao AY, Yang J, et al. The volume-regulated anion channel LRRC8C suppresses T cell function by regulating cyclic dinucleotide transport and STING-p53 signaling. Nat Immunol. 2022;23:287–302. 35105987 10.1038/s41590-021-01105-x PMC8991407

[54] Domizio JD, Gulen MF, Saidoune F, Thacker VV, Yatim A, Sharma K, et al. The cGAS-STING pathway drives type I IFN immunopathology in COVID-19. Nature. 2022;603:145–51. 35045565 10.1038/s41586-022-04421-w PMC8891013

[55] Huang L, Li L, Lemos H, Chandler PR, Pacholczyk G, Baban B, et al. Cutting edge: DNA sensing via the STING adaptor in myeloid dendritic cells induces potent tolerogenic responses. J Immunol. 2013;191:3509–13. 23986532 10.4049/jimmunol.1301419 PMC3788571

[56] He G, Dhar D, Nakagawa H, Font-Burgada J, Ogata H, Jiang Y, et al. Identification of liver cancer progenitors whose malignant progression depends on autocrine IL-6 signaling. Cell. 2013;155:384–96. 24120137 10.1016/j.cell.2013.09.031 PMC4015514

[57] Mauer J, Denson JL, Brüning JC. Versatile functions for IL-6 in metabolism and cancer. Trends Immunol. 2015;36:92–101. 25616716 10.1016/j.it.2014.12.008

[58] Sonnenblick A, Salgado R, Brohée S, Zahavi T, Peretz T, Van den Eynden G, et al. p-STAT3 in luminal breast cancer: integrated RNA-protein pooled analysis and results from the BIG 2-98 phase III trial. Int J Oncol. 2018;52:424–32. 29207087 10.3892/ijo.2017.4212 PMC6903891

[59] Binai NA, Damert A, Carra G, Steckelbroeck S, Löwer J, Löwer R, et al. Expression of estrogen receptor alpha increases leptin-induced STAT3 activity in breast cancer cells. Int J Cancer. 2010;127:55–66. 19876927 10.1002/ijc.25010

[60] Wakefield A, Soukupova J, Montagne A, Ranger J, French R, Muller WJ, et al. Bcl3 selectively promotes metastasis of ERBB2-driven mammary tumors. Cancer Res. 2013;73:745–55. 23149915 10.1158/0008-5472.CAN-12-1321

[61] Bachelot T, Ray-Coquard I, Menetrier-Caux C, Rastkha M, Duc A, Blay JY. Prognostic value of serum levels of interleukin 6 and of serum and plasma levels of vascular endothelial growth factor in hormone-refractory metastatic breast cancer patients. Br J Cancer. 2003;88:1721–6. 12771987 10.1038/sj.bjc.6600956 PMC2377148

[62] Salgado R, Junius S, Benoy I, Van Dam P, Vermeulen P, Van Marck E, et al. Circulating interleukin-6 predicts survival in patients with metastatic breast cancer. Int J Cancer. 2003;103:642–6. 12494472 10.1002/ijc.10833

[63] Kaplan RN, Riba RD, Zacharoulis S, Bramley AH, Vincent L, Costa C, et al. VEGFR1-positive haematopoietic bone marrow progenitors initiate the pre-metastatic niche. Nature. 2005;438:820–7. 16341007 10.1038/nature04186 PMC2945882

[64] Zhang Z, Karthaus WR, Lee YS, Gao VR, Wu C, Russo JW, et al. Tumor microenvironment-derived NRG1 promotes antiandrogen resistance in prostate cancer. Cancer Cell. 2020;38:279–96.e279. 32679108 10.1016/j.ccell.2020.06.005 PMC7472556

[65] Sohara Y, Shimada H, Scadeng M, Pollack H, Yamada S, Ye W, et al. Lytic bone lesions in human neuroblastoma xenograft involve osteoclast recruitment and are inhibited by bisphosphonate. Cancer Res. 2003;63:3026–31. 12810621

