# Supplementary material for: Therapeutic targeting of STING-IL6/STAT3 axis to inhibit osteoclastic niche formation and breast cancer bone metastasis
Source: Cell Death Discov. 2025 Oct 24;11:483. doi: 10.1038/s41420-025-02776-3 (PMC12552607; doi:10.1038/s41420-025-02776-3)
Supplement: Supplementary file 1 — Figures S1–S5 [file 41420_2025_2776_MOESM1_ESM.docx]

**Therapeutic Targeting of STING-IL6/STAT3 Axis to Inhibit Osteoclastic Niche Formation and Breast Cancer Bone Metastasis**


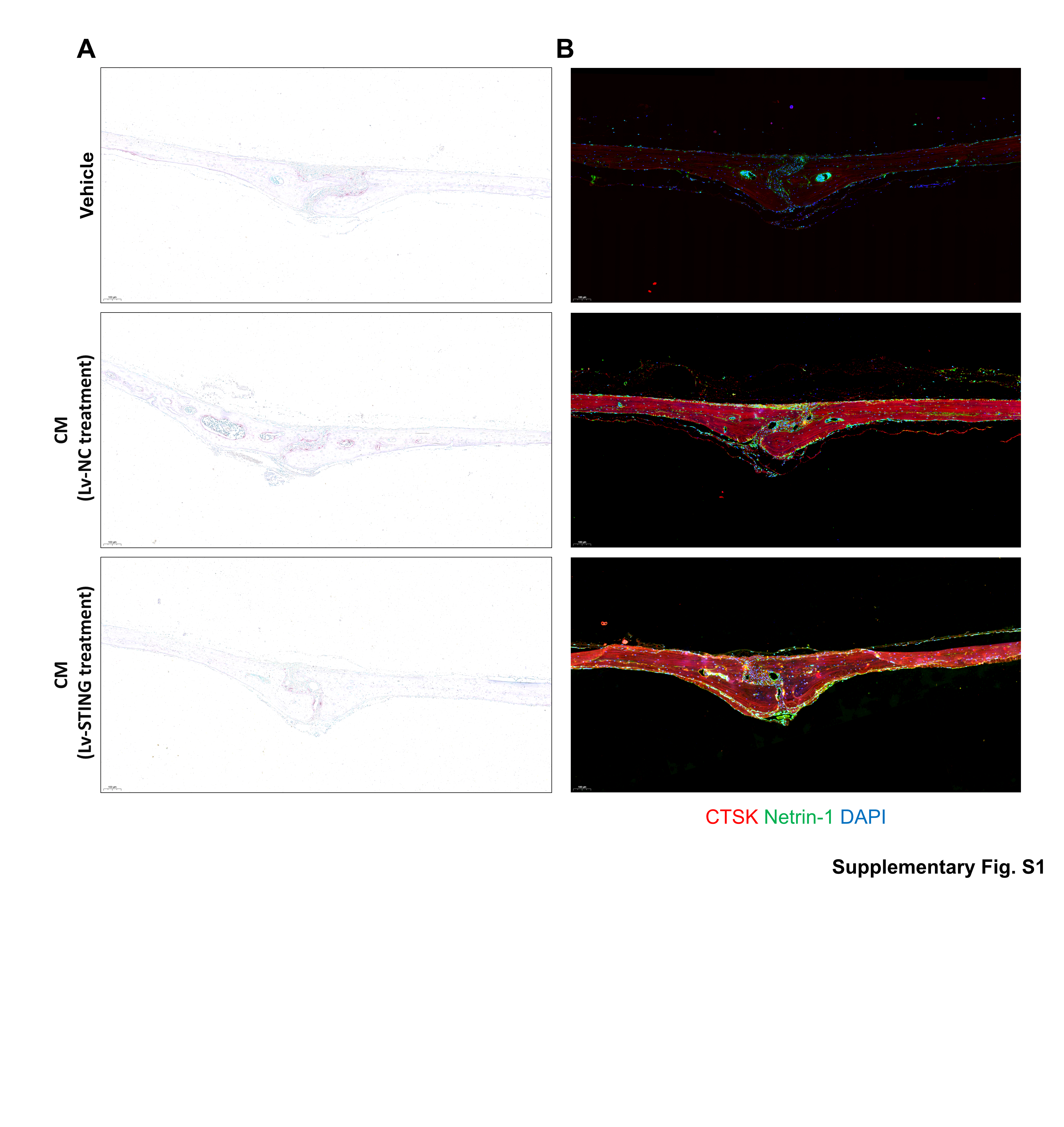


Supplementary Fig. 1 Conditioned medium derived from tumor cells overexpressing STING cannot improve the formation of the pre-osteoclastic microenvironment for cranial bone metastasis.


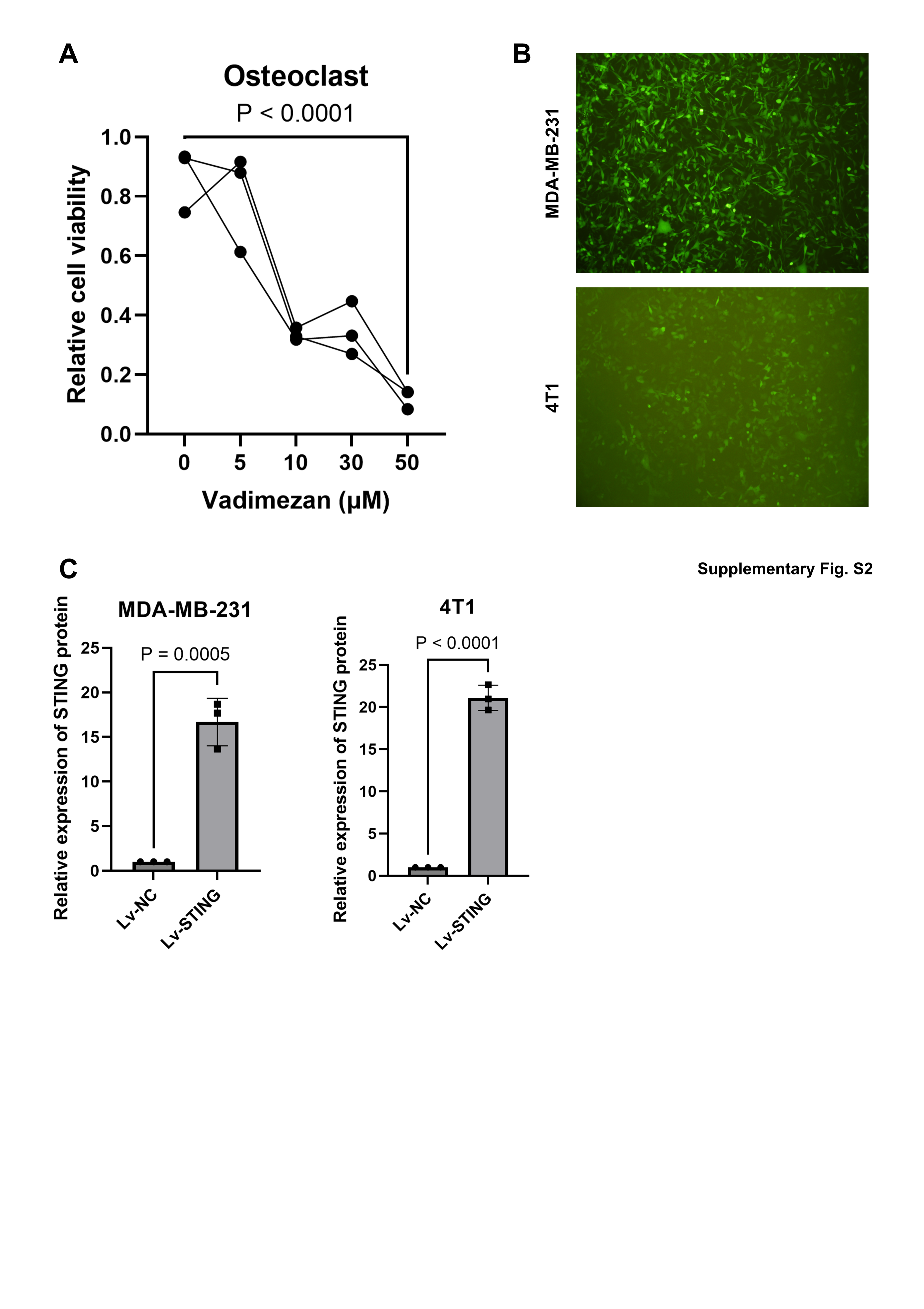


Supplementary Fig. 2 (a), The effects of varying concentrations of DMXAA treatment on osteoclast viability. (b), Microscopic confirmation of successful lentiviral infection leading to overexpression in MDA-MB-231 and 4T1 cells. (c) The relative amount of STING secretion in the tumor cell supernatant was evaluated by ELISA kit.


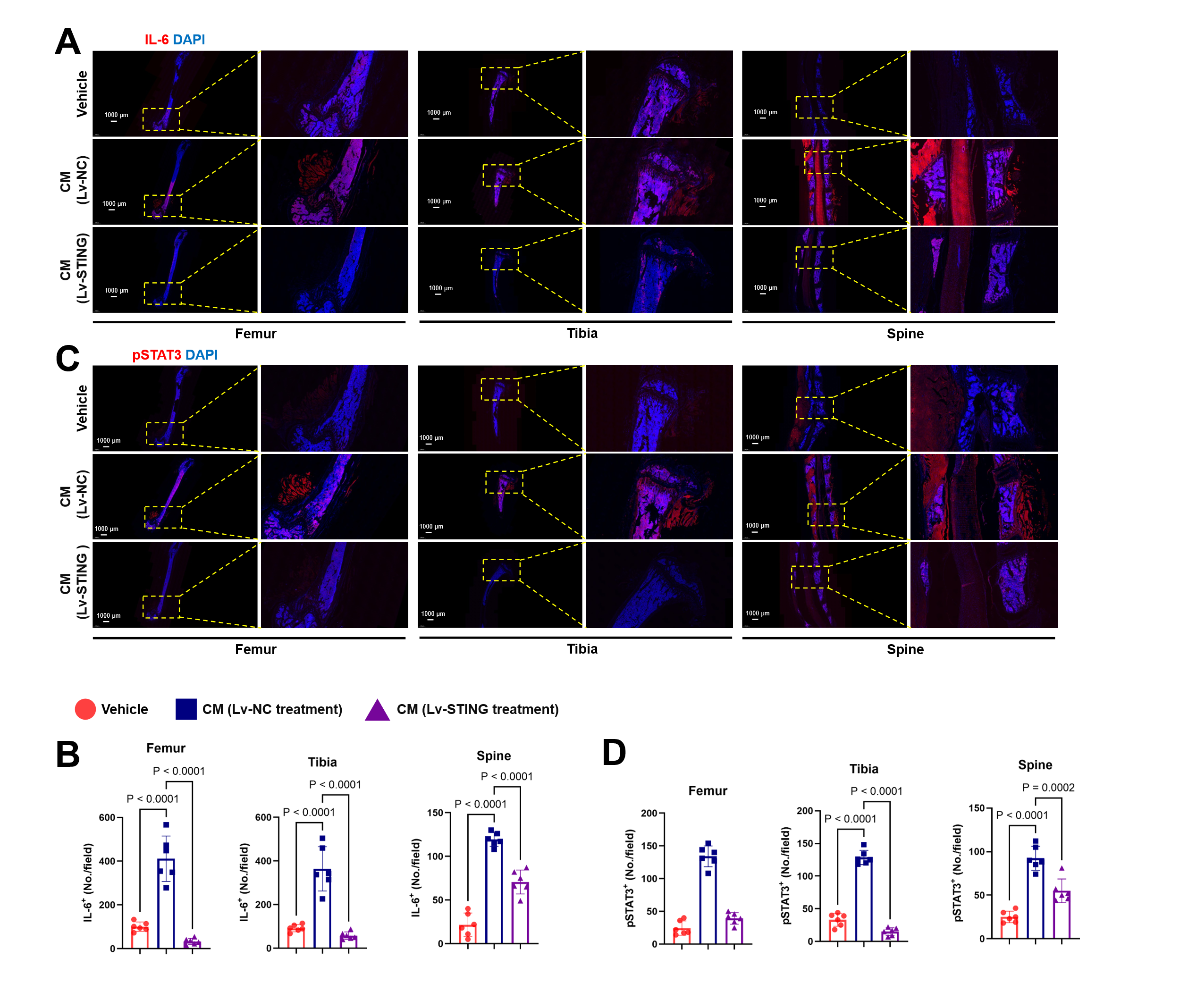


Supplementary Fig. 3 (A, B), Representative images of immunofluorescence staining of IL-6 (red) in the femur, tibia, and spine (top) and quantitative analysis (bottom). Scale bars, 1000 µm. n = 6 per group. (C, D), Representative images of immunofluorescence staining of pSTAT3 (red) in the femur, tibia, and spine (top) and quantitative analysis (bottom). Scale bars, 1000 µm. n = 6 per group. Data are presented as mean ± SD. Significance levels are indicated on top of each comparison. P values were calculated using a one-way ANOVA with a multiple comparisons test.


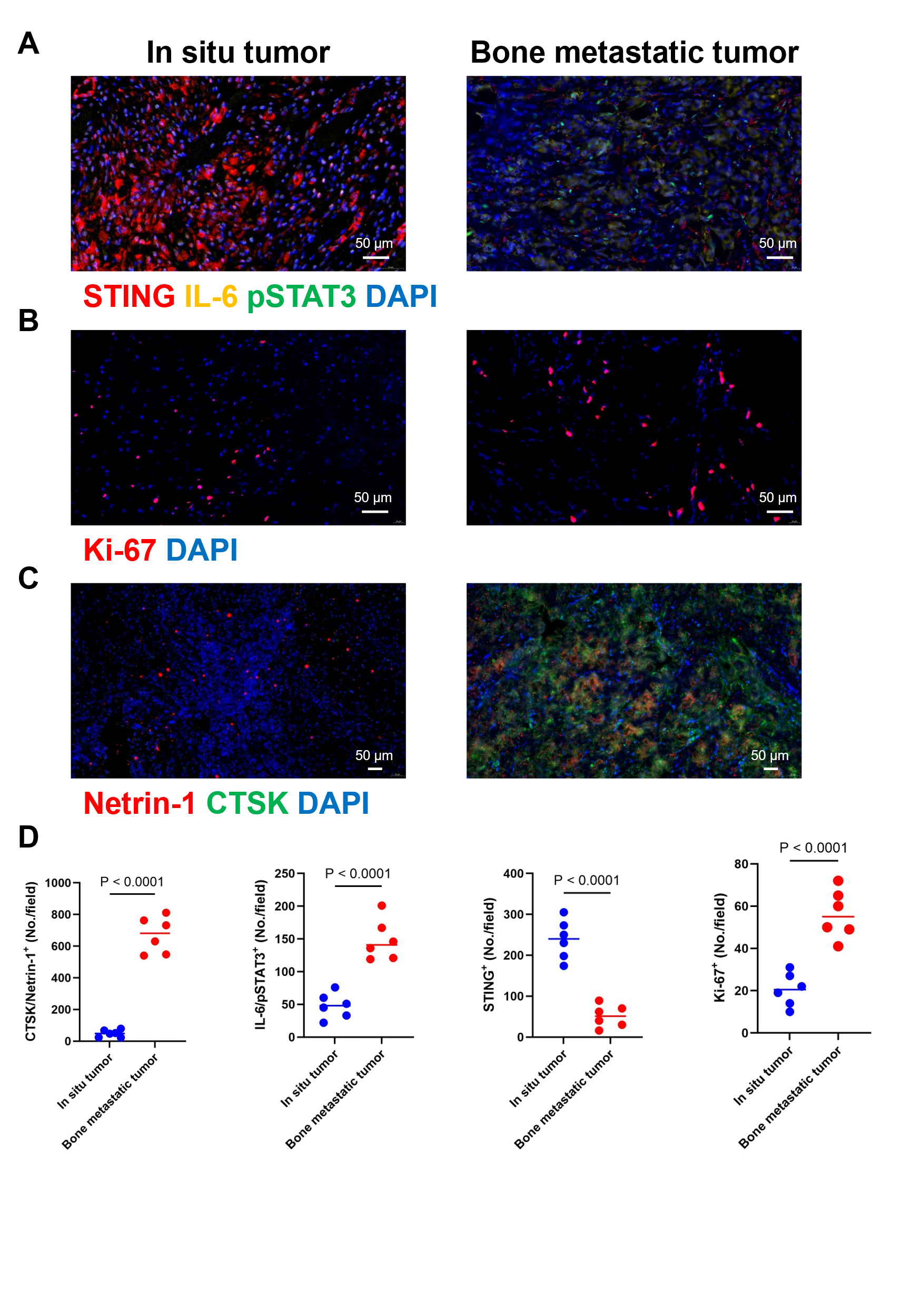


Supplementary Fig. 4 Comparison of breast cancer in situ and bone metastasis. (A) Representative immunofluorescence images showing staining of STING (red), IL-6 (yellow), and pSTAT3 (green). Scale bars, 50 µm. (B) Representative immunofluorescence images showing staining of Ki-67 (red). Scale bars, 50 µm. (C) Representative immunofluorescence images showing staining of Netrin-1 (red) and CTSK (green). Scale bars, 50 µm. Each group contained six samples. (D) Comparisons between the two groups were performed using an unpaired two-sided Student's t-test.


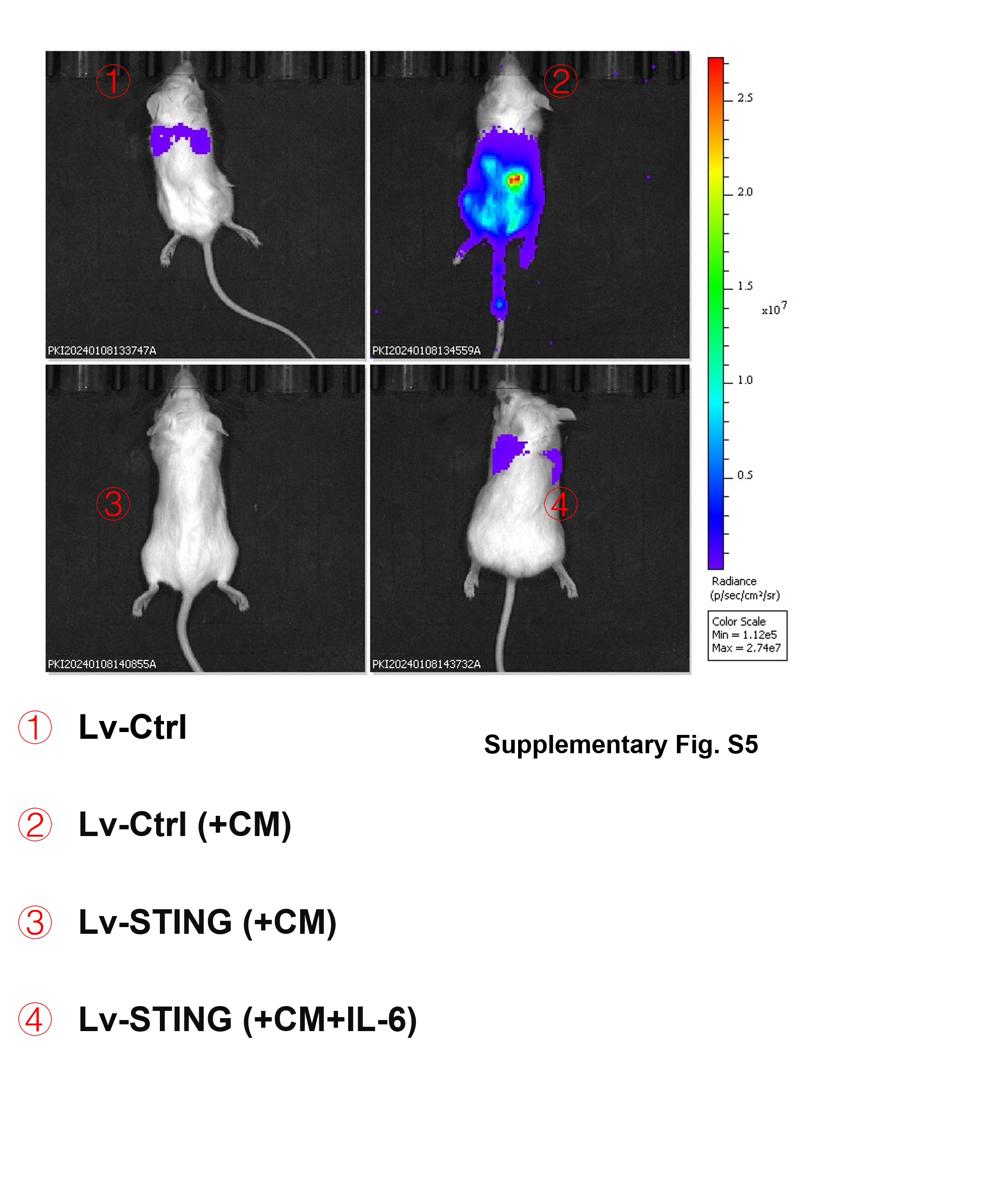


Supplementary Fig. 5 In vivo bioluminescence imaging showing tumor growth and metastasis.
