# Supplementary material for: Therapeutic targeting of STING-IL6/STAT3 axis to inhibit osteoclastic niche formation and breast cancer bone metastasis
Source: Cell Death Discov. 2025 Oct 24;11:483. doi: 10.1038/s41420-025-02776-3 (PMC12552607; doi:10.1038/s41420-025-02776-3)

**Therapeutic Targeting of STING-IL6/STAT3 Axis to Inhibit Osteoclastic Niche Formation and Breast Cancer Bone Metastasis**


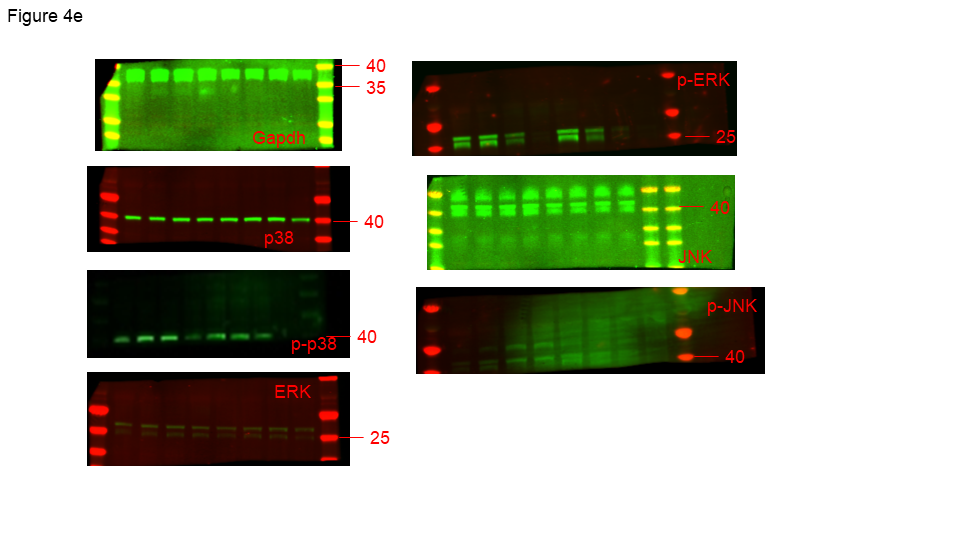


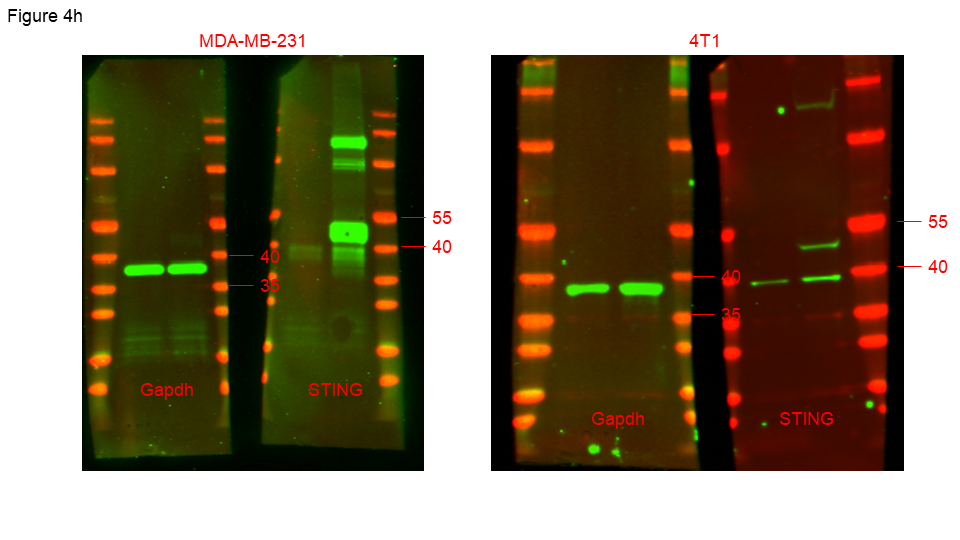

Supplement: Supplementary file 2 — The full uncropped Gels and Blots image(s) [file 41420_2025_2776_MOESM2_ESM.docx]
